# Unveiling bast fiber production in Upper Paleolithic North China: Microfibers and usewear traces on stone tools from Shizitan

**DOI:** 10.1371/journal.pone.0346767

**Published:** 2026-04-13

**Authors:** Li Liu, Yahui He, David Cohen, Xingcan Chen, Jinming Shi, Yanhua Song

**Affiliations:** 1 Department of East Asian Languages and Cultures, Stanford University, Stanford, California, United States of America; 2 Stanford Archaeology Center, Stanford University, Stanford, California, United States of America; 3 Department of Anthropology, National Taiwan University, Taipei, Taiwan; 4 Institute of Archaeology, Chinese Academy of Social Sciences, Beijing, China; 5 Shanxi Museum, Taiyuan, China; 6 School of Archaeology and Museology, Shanxi University, Taiyuan, China; University of Haifa, Zinman Institute of Archaeology, ISRAEL

## Abstract

Fiber technology—including the making of cordages, mats, baskets, and textiles—holds a crucial place in human history. However, uncovering archaeological evidence of early fiber products proves challenging due to their rapid decay. To address preservation hurdles, we employ a multi-disciplinary approach to interpret microfiber remains, drawing on microfossil remains, usewear traces, ethnographic observation, and experimental archaeology, to study artifacts from two Upper Paleolithic Shizitan (SZT) site localities on the North China Loess Plateau, dating 28,000–18,000 cal BP, encompassing the Last Glacial Maximum (LGM), on which we identify microremains of hemp and flax. Analyses of microfossil remains (microfibers, phytoliths, and fungi) and usewear traces on stone tools potentially reveal stages of bast fiber production, such as cutting stalks, retting, pounding fiber ribbons, and scraping to remove impurities. Such pounding and scraping are commonly associated with textile production in ethnographic accounts, and parallel evidence has also been observed on Neolithic stone tools in North China. Observations of colored fibers suggest SZT people may have extracted plant-based dyes and hematite pigment to color fibers. The cold-dry conditions of the LGM, which likely led to the depopulation of regions north of SZT, also may have driven increased fiber production, aligning with previously recognized shifts toward microblade production, broader interregional interactions, ritual activities, and broad-spectrum subsistence, including early wild millet use. This research provides new evidence for the deep history of fiber production in Upper Paleolithic China and demonstrates the value of usewear and microfossil analyses for studying ancient fiber technology.

## 1. Introduction

Fiber technology, including making cordage (strings, ropes, nets), mats, baskets, and textiles, holds a crucial place in human history [1,2]. Although this technology likely originated during the Paleolithic period, direct artifactual evidence from this era is exceedingly rare due to the rapid decay of organic fibers. The earliest known direct evidence of cordage macroremains is a 3-ply bast fiber cord fragment found on a stone tool attributed to Neandertals at Abri du Maras, France, dating to 50,000 BP [3]. Upper Paleolithic examples include three twisted fiber fragments, interpreted as remnants of cords, from Ohalo II, Israel (ca. 19,000 BP) [4]; and a heavy cord, twisted from three two-play fiber strings, found as a fossilized rope fragment in Lascaux Cave, France (ca. 17,000 BP) [2]. With such limited direct remains, researchers must primarily rely on indirect evidence to study fiber technology, such as artistic representations and tool types potentially associated with fiber production or cloth making [5,6], as well as usewear traces on tools indicating fiber processing, cordage making, or textile manufacturing and use [7,8]. A recent study combining morphological and spectroscopic analyses provided direct evidence for the processing of woad (*Isatis tinctoria*) leaves on grinding stones excavated from the Upper Paleolithic Dzudzuana Cave in Georgia, in the Caucasus region, 34–32,000 years ago. This plant is known as a source of indigotin, the blue pigment used for dye production; however, whether it was employed as a colorant, a medicinal substance, or both remains uncertain [9].

This paper concerns itself with identifying the processing of bast fibers in Upper Paleolithic North China, as bast fibers are the soft, flexible, strong fibers that traditionally have been used for producing cordage and textiles. In recent years, the use of compound light microscopes has proven successful for recognizing bast microfibers in sediments or on artifacts from Upper Paleolithic sites in Georgia, Israel, and China [10–13]. Microfibers are included among the microfossils classified as non-pollen palynomorphs (NPP), and NPP analysis offers significant potential for reconstructing ancient human activities related to fiber production and use [14–17]. However, the mere presence of bast fibers in sediments or on artifacts only offers circumstantial evidence of fiber production, providing little insight into the methods or locations of manufacture. This research aims to bridge this gap in the literature.

China has a rich history of fiber production, which, before the introduction of cotton during the historical era, was primarily centered on various types of bast fibers. Archaeologically, the earliest bast fiber products (macroremains), including cordage and textiles, have been found at a few Neolithic sites, mainly in water-logged conditions in southern China, dating to ca. 8000–5500 cal BP. The fibers used have been identified as ramie (*Boehmeria nivea*), velvetleaf (*Abutilon theophrasti*), kudzu vine (*Pueraria montana*), and hemp (*Cannabis sativa*) [18]. These four indigenous bast fiber species later become the most prevalently employed for fiber production in ancient China, with hemp, ramie, and kudzu fibers commonly used for both textiles and cordage, and velvetleaf mainly was used for cordage [19:277–278, 20:53]. China reportedly harbors nine native wild flax species (*Linum* spp.), primarily found in the north and west [21], but no macroremains of these species have been identified in the archaeological record.

The absence of macrofiber remains prior to the Neolithic period in China poses challenges to comprehending the early development of fiber technologies during the Paleolithic era. However, an NPP analysis of materials from the Upper Paleolithic site of Shizitan 29 in North China identified microfibers [11], offering new pathways for further research.

In addition, the recent integration of microfossil analysis and usewear studies on stone tools from the Peiligang site in Henan provides evidence of bast fiber production at a site of the same time period as Shizitan. At Peiligang, some microblades from Upper Paleolithic contexts (ca. 26,000 cal BP) exhibit traces of processing bast fibers, as indicated by usewear traces and microfiber residues on the tools [13]. In later deposits (ca. 8000 cal BP) at Peiligang, a set of tools associated with fiber production was discovered in two adjacent early Neolithic burials. Residue and usewear analyses suggest that this tool assemblage included a sickle for cutting fibrous plants, a pestle for pounding bast fibers, a scraper for extracting fibers, and a bone needle for sewing with bast fiber threads. Furthermore, the presence of microfibers on the surfaces of a human arm bone and on leg bones in one of these early Neolithic burials offers evidence potentially to support the presence of textile manufacture [13].

Similarly, at the late Neolithic site of Shimao in northern Shaanxi (ca. 4000 cal BP), an early urban center on the Loess Plateau, an assemblage of stone tools associated with bast fiber-textile production—including sickles, pounding tools, and scrapers—has been identified, along with textile fragments in cultural deposits and microfibers found on human skeletons [22,23].

These findings suggest a long-standing tradition of fiber processing that may extend back to a period preceding the Neolithic. In an attempt to illuminate the early history of this technology, this study delves into how aspects of an Upper Paleolithic fiber production process are potentially indicated by microfiber remains and associated usewear traces on stone tools at two Upper Paleolithic localities at the Shizitan site, SZT14 and SZT29 (SZT = the abbreviation for Shizitan, followed by the locality number).

## 2. Archaeological materials

### 2.1 Shizitan site and its environment

**Fig 1:**
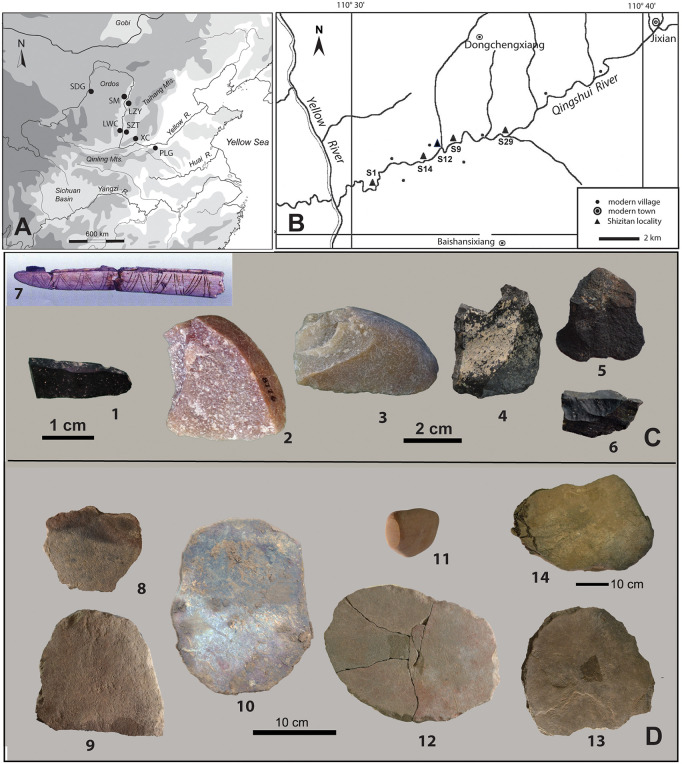
SZT site location and representative stone tools analyzed in this study. **(A)** Sites discussed in this study, SZT: Shizitan; LWC: Longwangchan; XC: Xiachuan; PLG: Peiligang; **(B)** Shizitan localities along the Qingshui River; **(C)** 1–6: SZT29 chipped stone tools (1: microblade [MB5]; 2: flake [SF4]; 3: flake [SF5]; 4: flake [SF8]; 5: flake [SF10]; 6: flake [SF23]); 7: composite sickle from Donghulin for comparison [31: plate 2:6]; **(D)** SZT 14 and 29 grinding stones (8: 29-GS1; 9: 29-GS3; 10: 29-GS5; 11: 29-GS7; 12: 29-GS8; 13: 29-GS13; 14: 14-GS3; all slabs but 11, a handstone).

The Shizitan site encompasses a cluster of several dozen Upper Paleolithic localities situated on the North China Loess Plateau along the Qingshui River, a tributary of the Yellow River, in Jixian, Shanxi province. Initially excavated in 1980 at what is now labelled SZT1, Shizitan was originally interpreted as a “Mesolithic” site [24]. However, subsequent fieldwork at multiple localities revealed that it primarily contains Late Upper Paleolithic (or LUP, defined by the presence of microblade pressure production) deposits. The dates for the overall occupation of the Shizitan site range ca. 28,000–8500 cal BP, with many localities flourishing during the Last Glacial Maximum (LGM; 27.54–23.34 Ka cal BP) [25] and continuing through the Late Glacial period. Among the identified localities, SZT1 [24], SZT5 [26], SZT9 [27], SZT12 [28], SZT14 [29], and SZT29 [30] have been excavated (Fig 1A and 1B).

Jixian today has a warm temperate continental climate, with an annual average temperature of 10ºC, an average annual rainfall of 580 millimeters, and a frost-free period of 172–180 days [32]. During the LGM, average temperature in the Shanxi region is estimated at 6.7–7.4 ºC lower than today [33]. During the Upper Paleolithic period, therefore, the Shizitan area experienced significant climatic fluctuations influenced by the LGM and subsequent warming trends, and these environmental dynamics likely influenced human adaptations.

The published paleoenvironmental reconstructions of the SZT29 site, based on pollen, NPP, soil micromorphology, and faunal remains from the site [11], along with an earlier sedimentological study of the Qingshui River terraces, including magnetic susceptibility and sporopollen analyses [34], provide details of changes in the site environment over time. In brief, they indicate that the region predominantly featured steppe and grassland vegetation, with LGM conditions marked by more arid, cold conditions. Layer 8, the lowest cultural layer of SZT29, dating prior to the LGM, ca. 28,000 cal BP and with Early Upper Paleolithic (or EUP) remains centering on an “advanced core and flake” industry that defines this period, featured a warmer and damper climate, with soil formation and deciduous trees in the region. LGM conditions are marked in Layer 7 (ca. 26–24 Ka cal BP) by increased *Pinus* and *Artemisia annua* pollen and *Equus caballus*/*E. hemionus* faunal remains, indicating the shift to a steppe or encroaching semi-desert environment. SZT Layers 6–4 (ca. 24–19.5 Ka cal BP) show that the cooling and drying trend continued, with dark coniferous species appearing and with *Artemisia annua* (sagebrush) and Chenopodiaceae pollen predominant among the herbaceous species, characteristic of a desert-steppe environment. Following these LGM conditions, the Qingshui River sporopollen assemblage indicates that as the generally ameliorating climate fluctuated, limited patches of deciduous broadleaf trees like birch (*Betula*) and oak (*Quercus*) expanded near the site region during relatively humid phases, interspersed within a generally mild and arid-to-semi-arid steppe environment [34]. During the Terminal Pleistocene period, overall conditions across North China became increasingly favorable for human occupation, which is supported by the re-inhabitation of regions further north than Shizitan that had been abandoned during LGM, such as at Shuidonggou [11,34,35] (for more detailed description see Supporting Information [SI], Section 1).

All of these SZT localities are primarily characterized by small flaked tools, grinding stones, and, after 26,000 cal BP, microblade pressure production technology. The absence of pottery, dwelling structures, human burials, or storage facilities suggests that the occupants were mobile hunter-gatherers [11,24,26,29,36].

The cold climatic conditions would have posed significant challenges to the survival of Paleolithic populations at Shizitan. Scholars have long proposed that Upper Paleolithic groups developed complex clothing systems—such as fitted or tailored garments including trousers with legs and shirts with sleeves, often worn in multiple layers—to provide enhanced protection against wind chill during the Last Ice Age. The habitual use of such garments likely prompted a shift in body decoration practices, from direct adornment on the skin to ornamentation applied to clothing. Archaeological evidence for this technological innovation includes bone awls and eyed needles used for sewing [37–40]. Additional evidence for decorated clothing is provided by beads that were sewn onto tailored garments [41]. The growing demand for complex clothing would, in turn, have increased reliance on fiber materials for sewing and stringing. This generalized scenario appears to be reflected in the artifacts uncovered at Shizitan, as discussed in more detail below (see Section 5.4).

### 2.2 SZT14 and SZT29 and previous functional studies of stone tools

The SZT14 and SZT29 localities were both situated directly on the northern bank of the Qingshui River, which is currently a narrow stream located approximately 50 m below SZT29 (Fig 1B; SI, Section 1; S1A–S1C Fig). However, during the site’s occupation period, the river was considerably elevated and broader, extending to a width of over 100 m [11]. Phytoliths from reed leaves (Phragmite bulliform) were found on stone slabs from SZT29, indicating the presence of shallow water bodies near the site [42].

The main occupations of SZT29 (36˚2′54″N, 110˚35′22″E) date ca. 28,000–18,000 cal BP. The excavations covered an area of 12,000 m^2^, with cultural deposits spanning a depth of 15 meters, delineated into eight cultural layers (SI, S1C Fig). These deposits revealed 285 hearth features and over 80,000 artifacts, including 74,735 stone artifacts. With its deep, well-stratified cultural deposits, and previously reported systematic series of 39 radiocarbon dates (for AMS dates see S1 Table) and paleoenvironmental reconstruction using the combined application of soil micromorphological, pollen and NPP, and faunal analyses [11], SZT29 provides the best dated record for the study of human adaptations across the LGM in North China. For this study, we divide the cultural deposits into four phases according to these previously reported stratigraphic changes and radiocarbon dating [11] as well as according to the major technological changes previously reported in lithic production, including the introduction and subsequent evolution of microblade technology [43,44] (Table 1):

**Table 1:** Chronology and archaeological contexts of SZT14 and SZT29 tools analyzed in this study.

Locality	Cultural Deposit Layer	Climatic Phase	Date (cal BP)	Grinding stones sampled	Stone flakes sampled	Microblades sampled	Total tools analyzed
SZT29	8	Phase 1: Pre-LGM	28,000–27,000	GS1*, GS2,	SF1, **SF10**		4
SZT29	7	Phase 2: Initial LGM	26,000–24,000	**GS3, GS4, GS5,** GS6, GS7	SF2, SF11, SF12	MB1, MB2, MB3, MB4, **MB5**, MB6	14
SZT29	6	Phase 3: Late LGM	24,000–19,500		SF13,	MB8	2
SZT29	5				**SF4, SF5**, SF14, SF15		4
SZT29	4			**GS8**	SF16, SF17, SF6		4
SZT29	3	Phase 4: Post-LGM	19,000–18,000		SF7, SF18, SF19		3
SZT29	2			**GS13**	**SF8**, SF20, SF21		4
SZT14	3 and 4	Phase 3: Late LGM	23,000–19,500	GS1, GS2, **GS3**			3
Total tools analyzed				12	19	7	38

*Artifacts in bold face indicate the 12 tools associated with fiber production identified in this study.

Phase 1 (Pre-LGM and EUP phase), consisting of Layer 8 and the base of Layer 7 with an advanced core and flake industry (ca. 28,000–27,000 cal BP);
Phase 2 (Initial LGM and LUP phase), represented by the top of Layer 7 and the first appearance of microblade pressure production (ca. 26,000–24,000 cal BP);
Phase 3 (Late LGM phase), encompassing Layers 6–4 (ca. 24,000–19,500 cal BP); and
Phase 4 (Post-LGM phase), corresponding to Layers 3 and 2 (ca. 19,000–18,000 cal BP) [11]. (For further details on the cultural deposits, including tabulations by phase of microremains, see SI, Section 1 text.)

SZT14 (110°32′40″E, 36°02′11″N) dates to ca. 23,000–18,000 cal BP. Excavations uncovered three continuous cultural layers within a depth of 10 m in an area of 25 m². The material remains include 1,643 lithic artifacts, 2,776 animal bone fragments, and 17 ash or burnt surface areas (fireplaces) [29,45]. The dates of the occupation of SZT14 are approximately contemporaneous with Phases 3 and 4 at SZT29 (ca. 24,000–18,000 cal BP, based on AMS ^14^C data [11,29]) (Table 1; for detailed SZT14 AMS dates see S2 Table).

Two previous studies by some of the authors involved sampling and examining three grinding stones from SZT14 Layers 4 and 3 (dating ca. 23,000–19,500 cal BP) (SI, S1 Table) and 40 lithic tools from SZT29 (comprising grinding stones, microblades, and small flake tools; dating ca. 28,000–18,000 cal BP) for usewear patterns and microbotanical remains (starch and phytoliths) (SI, S2A and S3 Figs, S3 Table). We also conducted experimental study of food processing using stone tools and analyzed the usewear on the utilized implements (S2: B, C). The usewear analysis on some of these tools revealed distinctive traces, including polish and fine striations, typical to tools used in processing siliceous plant materials. The microbotanical analysis of these tools also identified starch granules from Triticeae and Panicoideae grasses, various tubers, as well as phytoliths from grasses, suggesting their use in processing vegetal materials [42,46].

Similar usewear patterns, characterized by high polish and fine striations, have been identified on grinding stones (slabs and handstones) from a later-dating Shizitan locality, SZT9 (ca. 13,800–8500 cal BP), where starch gains from various starchy plants were also recovered [47]. Additionally, a study of flaked tools with usewear traces from SDG2 in the Shuidonggou Site Complex, Ningxia (ca. 33,000 cal BP [AMS ^14^C]–20,300 BP [OSL]), similarly identified starch grains associated with these tools [48]. These findings provide further evidence for the use of stone tools in plant processing.

### 2.3 Fibrous plants and fiber production in ancient China

The presence of high polish and fine striation traces on lithic tools can be attributed to processing silica-rich plants [49]. An experimental study confirmed that cutting wild millet grasses generates such usewear traces [50]. However, similar polish and fine striations can result from processing fiber-producing species, as well, as some bast fibrous plants contain silica in their epidermal cells. Such usewear patterns associated with fibers have been observed on flakes from Southeast Asia [51], flint implements from Europe [52], grinding stones from the Near East [53], as well as on Upper Paleolithic tools in North China, including microblades at Peiligang and flaked tools at SDG2 [13,48]. Therefore, the SZT stone tools might have played a multifunctional role in not only collecting food plants (wild grasses and tubers) but also in processing fibrous plants to produce fiber products.

Among China’s native fibrous plants, ramie, velvetleaf, and kudzu all exhibit ecological preferences characteristic of temperate to subtropical climates, favoring relatively warm conditions and substantial moisture [19,54,55]. Consequently, these species are unlikely to have survived near SZT during the LGM. The cold-resistant fibrous plants that may have thrived on the Loess Plateau of northern China during the LGM were likely limited to hemp and flax.

Hemp (*Cannabis* spp.) exhibits broad ecological tolerance and is distributed across temperate regions, including cold and dry continental environments. It is widely regarded as having originated in the temperate steppe zones of Central Eurasia and as having persisted in temperate refugia during glacial periods, demonstrating clear adaptation to cold continental climates [56: 84–98]. Flax (*Linum* spp.) likewise is capable of germinating at low temperatures and tolerating near-freezing conditions during early growth, and is commonly cultivated in cool climates with short growing seasons [57,58]. Given these well-documented ecological characteristics, there is no climatic barrier to the presence of wild hemp and flax in the Qingshui River valley during the Last Glacial Maximum. The reconstructed LGM environment of the Shanxi Loess Plateau—characterized by cold, open steppe or forest-steppe conditions and mean annual temperatures approximately 6.7–7.4 °C lower than today [33]—falls within the ecological tolerance ranges of both taxa.

Subfossil pollen records indicate that wild *Cannabis* originated on the northeastern Tibetan Plateau of northwest China by 19.6 million years ago, spreading eastward to eastern China by 1.2 million years ago. It remained continuously distributed across North China throughout the Pleistocene and Holocene, persisting through climate fluctuations. The plant appears to have been particularly well adapted to steppe environments, where it coexisted with *Artemisia* and Poaceae, demonstrating resilience under dry and temperate conditions [59]. A recent genetic study likewise supports an East Asian origin for domesticated *Cannabis,* suggesting its initial domestication around 12,000 years ago in China [60]. Together, these studies support the presence of *Cannabis* in northern China during the occupation period of SZT.

The earliest evidence of flax fiber use comes from Dzudzuana Cave in Georgia, dating to around 30,000 years ago, indicating a long history of flax utilization [10], although this identification remains debated [61,62]. One species of wild flax (*Linum angustifolium* Huds.) is the progenitor of domesticated flax (*L. usitatissimum*) and has a broad biogeographical range spanning western Europe, North Africa, western and southern Asia, and the Caucasus region [63], but it has not been reported in East Asia. In China, however, there are nine native wild flax species, distributed mainly in the northern and western regions [21], suggesting adaptation to dry and cold climates, but their prehistoric distributions remain largely unstudied, and there is no evidence of domestication of native wild flax species in China.

In China, a hemp seed slightly larger than the typical wild hemp was recovered from the early Neolithic Zhuzhai site near Zhengzhou, Henan, dating to ca. 7900–7600 cal BP [64]. This early Neolithic presence of an enlarged hemp seed supports a long history of hemp utilization in the region and aligns with the pre-Neolithic domestication date estimated through genetics [60]. Furthermore, hemp and flax seeds have been found at late Neolithic Shimao and at Linzheyu in northern Shanxi, about 250 km north of SZT, dating to ca. 4300–3800 cal BP [65,66]. While wild flax species remain widely distributed in northern China today, domesticated hemp has largely replaced its wild counterpart, as indicated by regional botanical records [19,67–69].

Well-adapted to dry and cold environments, hemp and flax have historically been utilized for fiber production, oil extraction, and medicinal purposes in China [21,68: 1-5, 69,70]. Given their ecological resilience, it is plausible that these fibrous plants were present during the relatively dry and cold climatic conditions in the Loess Plateau region during the Upper Paleolithic period. Significantly, the NPP analysis of sediment samples from SZT29 identified microfibers from hemp and flax, with numerous fibers displaying shades of pink, blue, black, and grey [11]. Furthermore, the presence of an eyed bone needle and numerous ostrich eggshell beads, some with wear traces, at SZT29 strongly indicate the use of thread or stringing [71,72].

To further investigate fiber technology at Shizitan, we reexamined the original residue assemblages from SZT14 and SZT29.

### 2.4 Residue sample processing

Samples from tools from SZT14 were collected in 2009–2010, and from those from SZT29 in 2009 and 2015. Residues were recovered either by applying distilled water to the tool surface and pipetting the solution, by removing adhering sediments, or by ultrasonic extraction (ultrasonic bath or toothbrush).

All samples were processed for starch and phytolith extraction using sodium polytungstate (specific gravity 2.35). Extracted residues were mounted on glass slides in 50% (vol/vol) glycerol and 50% (vol/vol) distilled water, and coverslips were sealed with nail polish. Slides were examined under a Zeiss Axio Scope A1 microscope with polarized light and differential interference contrast (DIC) optics. Images were captured using a Zeiss Axiocam HRc digital camera with Zeiss Axiovision software (Version 4.8).

After initial analysis, the slides were stored in Heathrow Scientific slide boxes on laboratory shelves at the Stanford Archaeology Center and were not reopened. At the time of reexamination for the present study, most slides had dried out.

Rehydration was carried out following a method suggested by Christine Hastorf (University of California, Berkeley; personal communication). Each slide was rinsed with distilled water, placed in a clean plastic Petri dish, and submerged in a minimal volume of distilled water sufficient to cover the slide. The dish was covered to reduce evaporation. Slides were monitored periodically as water gradually penetrated beneath the coverslip. After rehydration, slides were removed and excess surface water was allowed to evaporate. Coverslips were then resealed with a fresh layer of nail polish prior to microscopic observation. The rehydration process typically required several hours or longer.

This simple rehydration procedure successfully restored 38 out of 43 slides for renewed analysis. The analyzed assemblage includes 12 grinding stones, seven microblades, and 19 small flake tools, with particular emphasis on the identification of fibers. The depositional contexts of all 38 tools are summarized in Table 1, and the associated residue findings are presented in S3–S7 Tables.

## 3. Research methods

Our multidisciplinary approach integrates microfossil analysis (including microfibers, phytoliths, and fungi), ethnographic investigations, and experimental replication of fiber processing using stone tools, followed by usewear and residue analyses of the experimental implements. In addition, we conducted a testing program to evaluate whether the residue samples had been affected by contamination from modern environments.

### 3.1 Microbotanical taphonomy

Microbotanical remains, including starch granules, phytoliths, and fibers, are frequently preserved in archaeological sediments and found on artifacts. Microfibers and starch granules are preserved in certain conditions, especially when they are quickly buried and thus less exposed to microorganisms and oxygen. Microparticles can also become embedded in crevices or on the surfaces of artifacts like pottery, stone tools, or grinding stones. Once trapped in these materials, they are protected from environmental exposure, physical wear, and biological decay, helping them survive over long periods [73,74]. Certain sedimentological conditions also favorably affect the preservation of these microremains, such as smaller aggregate size, which reduces the destructive enzymatic activities induced by fungi and bacteria [75]. While understanding preservation is important, we must also strive to understand the anthropogenic and geogenic formation processes that lead to the deposition and potential re-deposition of microfibers at the site.

At Shizitan, microfibers discovered on archaeological tools and in the sediments could derive from several potential sources. Firstly, if the tools were used in fiber processing, some microfibers and associated elements (e.g., epidermis, crystals, hair cells from fibrous plants) may have adhered to their surfaces. Secondly, microfibers could be present from human usage of fiber-based products in the vicinity that released particles that may have become affixed to nearby stone tools or became deposited in the site surface. These microremains would be contemporaneous with the tools and the archaeological layer and thus still be able to provide insights into the anthropogenic origins of certain ancient fiber-related materials, including dyed fibers. Thirdly, dyed fibers discovered on harvesting tools could originate from the fiber products associated with the individuals who used the tools, rather than being resultant from their production using the tools. While these fibers may be deemed as “ancient contamination,” it is crucial to recognize their anthropogenic nature—that they derive from human activities during the time of the site’s occupation. Lastly, certain post-depositional processes, including the deposition of waterborne microfibers and bioturbation, could result in the presence of fibers in the sediments [e.g., 17]; however, no evidence of this scenario has been identified at Shizitan.

### 3.2 Methods for identifying fiber production tools

The methods employed in an initial study of SZT lithic tools, involving usewear and microbotanical (starch and phytolith) analyses, which were previously published [42,46], are summarized in SI, Section 2. For the fiber analysis, chemical analysis was considered; however, it was not applied due to several methodological constraints. First, the microfibers identified in this study occur at the micrometer scale and in extremely limited quantities. Importantly, these remains were already mounted on permanent microscope slides under coverslips during the analytical process. As a result, it is not physically feasible to reopen the slides and extract individual fibers for additional chemical analyses without risking complete loss or contamination of the material. Second, even if extraction were possible, many chemical techniques (e.g., FTIR, GC–MS, Py-GC/MS) are inherently destructive or require larger amounts of relatively clean material than are available in these samples [76]. Third, the chemical composition of plant fibers is dominated by cellulose, hemicellulose, and lignin, which are broadly similar across taxa. Spectroscopic studies have shown that cellulosic fibers often exhibit highly similar chemical signatures, making taxonomic differentiation difficult [77]. Given these limitations, microscopic morphological analysis remains the most effective and widely adopted method for identifying archaeological plant microfibers, particularly at this scale [10,11]. This approach also enables direct integration with other microfossil evidence (e.g., starch granules, phytoliths, and fungal remains) observed on the same slides.

For the current fiber analysis, the examination of the residue samples was performed using a Zeiss Axio Scope A1 transmitted light microscope equipped with polarizing filters at magnifications of 100 × , 200 × , and 400 × . Images were captured using Zeiss Axiocam HRc digital cameras and Zeiss Axiovision software Version 4.9.1. To analyze the fibrillar orientation, we employed a Nikon Eclipse E200 microscope equipped with polarizers and a red tint plate (530-nm full wave compensator) for the modified Herzog test. Images were captured using Pixe LINK PL-D775 UC digital camera software at a magnification of 100× (see SI, Section 2.3 and S4 Fig for details).

**Fig 2:**
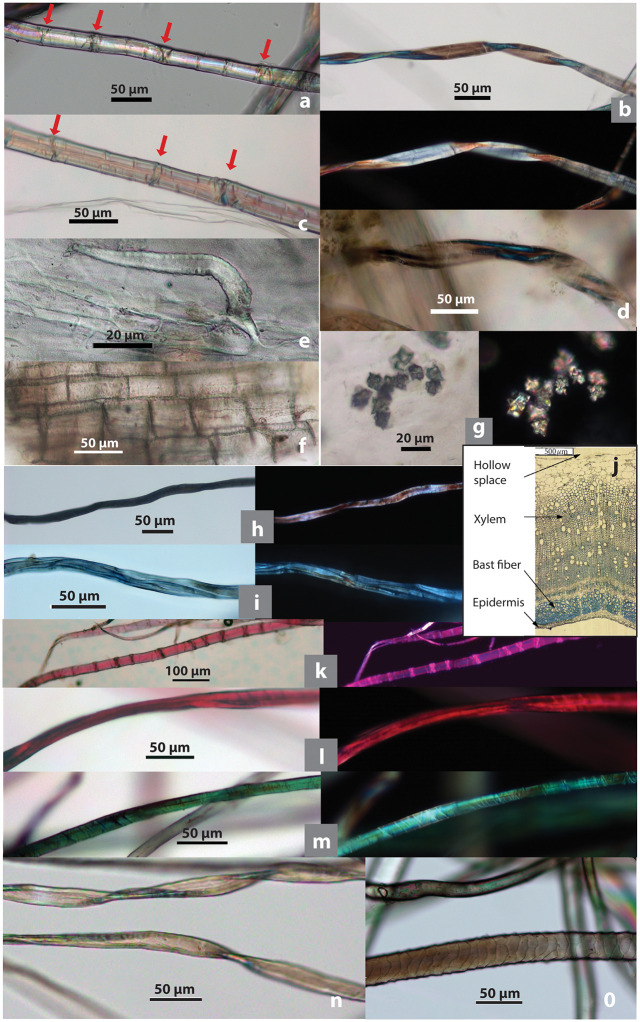
Modern microfibers and associated elements as comparative references. **(a)** Hemp showing dislocations (indicated by arrows); **(b)** damaged hemp showing twisted ribbon form; **(c)** flax, showing dislocations (indicated by arrows); **(d)** flax showing twisted ribbon form; **(e)** hemp hair cell; **(f)** hemp pitted vessel cells; **(g)** hemp clustered crystals; **(h)** dyed bast fiber, black; **(i)** dyed bast fiber, blue; **(j)** hemp stem cross section; **(k)** dyed bast fiber, pink; **(l)** dyed bast fiber, red; **(m)** dyed bast fiber, green; **(n)** cotton; **(o)** synthetic fiber (upper) and wool (lower). (b, g, h, i, k-m show brightfield and polarized views of the same image; plant and wool samples collected from China by Li Liu; [j] adapted from [78: fig 3]).

The stem of fibrous plants is composed of multiple layers, including the xylem, phloem, and epidermis (Fig 2j). Bast fibers, obtained from the phloem, are cellulosic fibers that necessitate specific extraction methods. The extraction process involves the removal of the epidermis and xylem tissue attached to the fibers [78–80]. Based on ethnographic observations in Asia [81–84], as well as our interviews with informants who have experience in bast fiber production in northern China (see S8 Table), the essential stages of traditional bast fiber production (especially for hemp) can be summarized as follows: (1) Cutting the fibrous plant stalks and stripping the leaves using sickles or knives. (2) Retting the stalks in water or in the field to aid in separating the fiber from the stem. This process dissolves or rots away much of the cellular tissues and gummy substances (pectins) surrounding bast fiber bundles by bacterial or chemical degradation. (3) Pounding the fibers with stone or wooden tools to enhance their softness, strength, and ease of twisting. (4) Scraping off the epidermis and coarse fibers using scrapers. (5) Splitting the fiber into narrower strips. (6) Splicing the strips together by hand to create continuous, long fiber strings. (7) Spinning the fibers by hand or employing other mechanisms to facilitate thread production. These fibers can then be utilized to make string, cordage, containers, and potentially textiles. They may also undergo dyeing processes.

**Fig 3:**
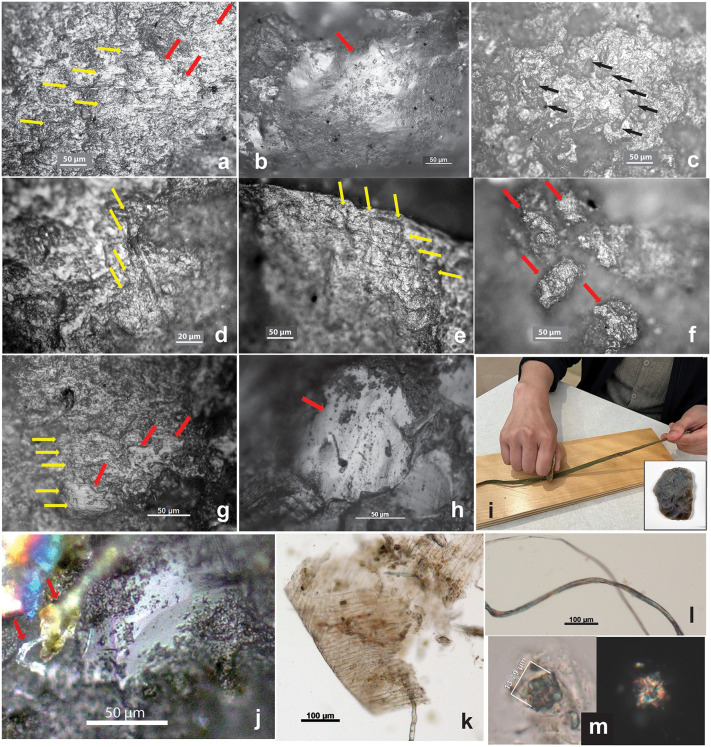
Usewear traces on SZT29 tools (a–f) compared with usewear and residue on experimental tools (g–m). **(a-f) SZT29 tools. (a)** Microblade (MB5), high polish (indicated by red arrows) and fine striations parallel to the edge (indicated by yellow arrows), compare with (g); **(b)** flake tool (SF4), high polish, compare with (h); **(c)** handstone (GS7), high polish with micropitting (indicated by black arrows); **(d)** flake scraper (SF5), fine striations perpendicular to the edge (indicated by yellow arrows); **(e)** flake tool (SF8), high polish and fine striations perpendicular and parallel to the edge (indicated by yellow arrows); **(f)** grinding slab (GS8), isolated polished areas (indicated by red arrows). **(g–m) Experimental tools. (g)** sandstone knife, cutting and scraping velvetleaf stems, 60 min, high polish and fine striations parallel to the edge (indicated by red and yellow arrows); **(h)** flint flake, scraping hemp ribbon, 30 min, high polish (indicated by red arrow); **(i)** scraping hemp ribbon with a chert flake shown in lower right; **(j)** microfibers attached to the chert flake after use; **(k–m)** epidermis, microfibers, and crystal found in the residue from the chert after use (hemp ribbons were sourced from Hemp Traders in Los Angeles, California).

Significantly, while Stages 3 and 4 (pounding and scraping the fiber ribbons) appear to be essential procedures in the traditional production of bast fiber textiles, as recorded for hemp textile making in Korea [81: figs 3,5] and Vietnam [85], these two steps typically are omitted when manufacturing cordage (string and rope) from hemp, ramie, or velvetleaf in northern China, as such products do not require fibers to be highly refined or softened. Based on our interviews with informants who process bast fibers in rural areas across three northern Chinese provinces – Henan, Shaanxi, and Qinghai – farmers have traditionally cultivated these plants for both food and fiber. The fibers are commonly used to produce string for stitching shoe soles and rope for various subsistence activities, but pounding and scraping procedures are not performed (SI, S8 Table). This ethnographic information suggests that different fiber-processing activities involve differing toolkits. Accordingly, the presence of tools associated with fiber preparation Stages 3 and 4 above—such as pounding implements and scrapers—together with concentrated fiber remains in the archaeological record could potentially indicate the possibility of textile manufacturing at a site.

With many of these fiber production activities involving tools, microfiber and associated elements from fibrous plants may adhere to the surfaces of the utilized tools. The harvesting and processing of fibrous plants also would likely cause specific usewear traces on these tools, and microfibers and associated elements could also become deposited in the sediments and on the objects near the activity areas. Additionally, the presence of shallow water bodies near the site, such as noted for the Shizitan localities, could be ideal for retting bast fibers. To test these propositions, we conducted experimental studies of processing various fibrous plants, as described below.

### 3.3 Usewear patterns and fiber remains on experimental tools

Previous usewear analyses of the SZT lithics indicate that some small flakes, microblades, and grinding stones were used to process soft, siliceous plants, as evidenced by the presence of high polish and fine striations [42] (Fig 3a–3f). Such usewear patterns are commonly observed on tools used to process plants with a high phytolith content, such as grasses [49,86]. However, they may also be associated with fiber processing [87,88], since epidermis in bast fibrous plants contains phytoliths.

We conducted a series of experiments, previously published [13], to investigate usewear patterns and fiber-related residues on stone tools after fiber processing. The experiments involved using a sandstone knife to cut velvetleaf stems (as a substitute for hemp stems, which were not available to us), as well as tools crafted from chert, fine sandstone, and slate to scrape hemp ribbons sourced from Hemp Traders in Los Angeles, California. Each tool was employed for a duration of 30–60 minutes, followed by usewear and residue analyses. The experimental scraping procedures are described below.

The dry hemp ribbons were first soaked in water for two days and then manually scraped with lithic tools (S5 Fig: 2, 3). After processing, the small chert flake was examined directly under a microscope, whereas the PVS method (polyvinyl siloxane peels examined under a microscope; discussed in SI Section 2) was applied to the larger artifacts. Images were captured at 200 × magnification. The results show that all stone tools developed a moderate to high degree of polish, often accompanied by fine striations (Fig 3g–3h), comparable to those observed on some SZT tools (Fig 3a and 3b). Higher levels of polish are especially evident on lithic materials with flat surfaces and high hardness (Mohs 6–7), such as chert. In addition, microfibers were observed on tool surfaces (S6 Fig), indicating that fiber residues can adhere to tools during processing.

**Fig 4:**
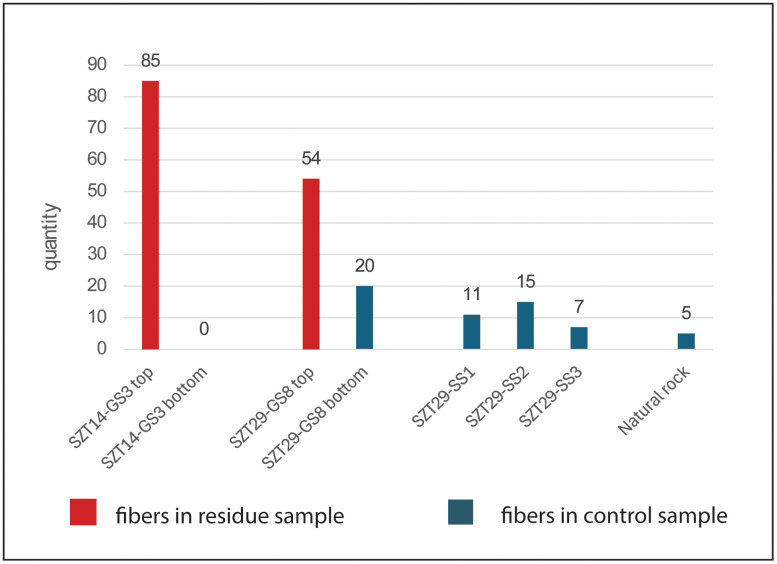
Fiber quantity comparison between residue and control samples. Control samples contain considerably fewer fibers than residue samples.

Another experiment involved scraping retted fiber ribbons with a stone tool. Bast fiber ribbons were retted in water at room temperature (approximately 22 °C) for 10 days, then scraped on a wooden board using a fine sandstone tool (S5 Fig: 4). This process effectively removed the epidermis. Residue analysis of the tool revealed abundant bast microfibers (mostly damaged), epidermal tissues, and crystalline particles (Fig 3i–3m).

Significantly, these wear patterns and fiber-associated materials in the residues align with those identified on several flake tools and sandstone slabs from SZT (Fig 3). These experiments confirm that on archaeological tools, the combined presence of notable concentrations of fiber remnants and high levels of similar polish and fine striations can serve as an indicator of the tool’s function in fiber processing.

### 3.4 Bast fiber identification

Bast fibers can be distinguished from other fiber types by their characteristic segmented structure. Under an optical microscope, bast fibers have a unique segmented morphology (nodes) with transverse dislocations or cross markings (Fig 2a and 2c). Cotton fibers are characterized by their flat, ribbon-like shape and irregular twisting; animal (proteinaceous) fibers exhibit scale patterns on the surface; and synthetic fibers generally show smooth and uniform surface and lack of cellular structure (Fig 2n and 2o) [89]. In comparison, pinpointing particular varieties of bast fibers can pose a greater challenge [90]. To identify bast fibers, we examined several morphological features using polarized and brightfield views under a compound light microscope. These features include (1) the segmented structure (dislocations and cross markings); (2) fibrillar orientations (Z-twist vs. S-twist) examined with modified Herzog test (or red plate test); (3) calcium oxalate crystals (clustered or solitary); and (4) cross-sectional diameters [89,91–93] (see SI, Section 2.4; S4 Fig for detailed explanation). The morphological characteristics of Chinese native fibers, including hemp, have been documented in detail in previous research [18], and these data are utilized in the present study (Fig 2).

In this study, we collected two fibrous plants from northern China as reference samples— hemp and flax (*Linum* sp.)—which are most likely to have been available in the study area during the Upper Paleolithic, as discussed above. Previous studies [18,79,92,94] demonstrate that several morphological characteristics can be observed and used as key features to identify fibers and associated elements:

Fibers: (1) Dislocations and cross markings: Hemp and flax fibers exhibit prominent dislocations and cross markings. (2) Fibrillar orientation: Hemp fibers show a Z-twist, while flax fibers exhibit an S-twist. (3) Damaged fiber form: Some damaged hemp (about 20%) and flax fibers (5%) tend to display twisted ribbon-like forms. (4) Cross-sectional diameters of hemp and flax are similar in maximum size range (6.02–37.06 µm and 16.33–44.84 µm, respectively) (Fig 2a–2d; Table 2).

**Table 2:** Major morphological characteristics of hemp and flax microfibers.

Fibrous plant	Dislocations and cross markings (n = 100)	Fibrillar orientation	Damaged fiber (n = 100)	Cross-section diameter (n = 100)	Crystals (n = 100) Size (n = 53)	Hair cell phytoliths
Hemp	Prominent	Z-twist	Twisted ribbon 20%	6.02–37.06 Avg. 20.50 µm	Mostly clustered, 2% solitary; size 8.96–29.86, avg. 11.96 µm	Narrow base, curved or hooked shape, rounded end
Flax	Prominent	S-twist	Twisted ribbon 5%	13.35–36.83 Avg. 26.73 µm	Absent	Absent

Associated elements: (5) Calcium oxalate crystals: Bast fibers contain two types of crystals, clustered and solitary; clustered crystals in hemp fibers measure 8.96–29.68 µm, whereas flax fibers contain no crystals. (6) Hair cells (a phytolith morphotype) associated with epidermis are curved in hemp, while flax lacks the typical form of hair cells altogether. (7) Pitted vessel elements are narrow in flax and wider in hemp (Fig 2e–2g; Table 1).

Not all of these fiber-associated elements may be present for each archaeological tool due to preservation conditions; however, when analyzed collectively as an assemblage, they enhance confidence in identifications [18].

### 3.5 Contamination assessment and control

To minimize airborne fiber contamination, strict laboratory cleaning procedures were implemented. All equipment and work surfaces were cleaned after each use, and the entire laboratory underwent routine weekly cleaning. Modern reference samples and archaeological samples were processed in separate facilities using distinct toolkits to avoid cross-contamination. In addition, the surfaces of mounted slides were cleaned with a compressed-gas duster prior to microscopic examination.

A potential source of microfibers is modern contamination that could occur during the post-excavation process and laboratory analysis, such as from the clothing worn by personnel. However, modern textiles predominantly utilize cotton, synthetics, or wool, while bast fiber clothing is extremely rare. This situation aids in separating modern contamination from ancient bast fibers.

In our analysis, we identified and recorded a very small number (n = 19) of fibers of cotton, synthetics, or wool. This represents only 3.5% of the total fiber count identified from the residues (n = 546, see below), indicating minimal modern fiber contamination. These fibers were subsequently excluded from further analysis due to their likely modern origin.

Another potential source of modern contamination is the use of paper labels, which are made of bast fibers, during the post-excavation process. Because the smaller tools were stored together in plastic bags along with paper labels, this might have introduced contaminants. This situation only applies to the small flakes and microblades, but not to the larger grinding stones from SZT. In the latter case, paper labels were sealed in plastic bags, isolated from the tools. To assess the potential for paper label contamination on small lithic tools, we conducted an experimental study replicating the post-excavation storage process as follows.

Firstly, a stone flake was thoroughly washed with an ultrasonic bath to remove any dust. Next, the clean flake and a paper label were placed together in a small plastic bag, and the bag was manually shaken for 30 seconds, causing the flake to rub against the label. The flake was then removed from the bag, rinsed with water in a new bag, and the water discarded. Subsequently, the flake was submerged in clean water in a plastic bag and shaken in an ultrasonic bath for 3 minutes. Finally, the water was centrifuged, the supernatant decanted, and the remaining solution mounted on a slide for examination. Two samples were processed, and each revealed two or three bast fibers. The results demonstrate that a minimal number of fibers (2–3) from a paper label could be transferred to a tool if stored together in a plastic bag. However, this experimental action is likely more vigorous than the actual situation, as the tool and paper label would have been stationary inside the bag for post-excavation storage.

Although there is a possibility of minimal transfer of microfibers from paper labels to the artifacts, the presence of a substantial concentration of fibers in the tool residues is unlikely to be attributed solely to modern paper contamination. Instead, it is more probable that these fibers are associated with the original function of the tool and/or the anthropogenic environment during its use. Furthermore, the identification of dyed bast fibers and the presence of specific microfossil elements related to bast fiber plants (such as crystals) further support the authenticity of the fibers. These characteristics are not typically found in modern paper labels, thus providing additional evidence of the fiber’s origin and association with the tool.

When assessing the presence of fiber remains from ancient times, it is crucial to consider two key factors: high concentrations of fibers and the presence of associated bast fiber elements in residue samples. Our focus is specifically directed towards tools that not only demonstrate high fiber concentrations but also exhibit usewear traces that are likely associated with the processing of fibrous plants. By considering these combined factors, we can identify direct evidence of ancient fiber remains and their potential significance in relation to the tool’s function and past human activities.

A previous study of SZT29 recovered microfibers and other NPPs in the site sediments suggestive of human activities at the site [11]. To examine differences in fiber deposition between sediments and fiber-working artifacts, we analyzed six control samples for fiber content: (1) A sample extracted from a heavily weathered, unused surface of a slab from SZT14 (14GS3:3) revealed no fibers, compared to 85 fibers on its used surface; (2) a sediment sample from an unused surface of a slab from SZT29 (29GS8-bottom) contained 20 fibers, compared to 54 on its used side; (3) three soil samples (29SS1–3) from SZT29 collected near slab 29GS8 contained a low quantity of fibers (n = 11, 15, 7); and (4) a natural sandstone sample from outside the site (SS1) contained five fibers, none of which were dyed. Notably, the quantities of microfibers in the control samples (n = 0–20) are in general much lower than those on the working surfaces of the artifacts (n = 6–108) (SI, S6 Table).

These results reveal a clear and consistent pattern: (1) unused tool surfaces contained significantly fewer fibers than their corresponding used surfaces; (2) sediment samples from within the site yielded fewer fibers than used tool surfaces; and (3) the natural rock sample collected outside the site contained the fewest fibers overall (Fig 4). Moreover, dyed fibers—indicative of deliberate human processing—were identified exclusively in the assemblages associated with artifacts and site sediments, whereas none were present in the natural rock sample (SI, S6 Table). The pronounced quantitative gradient and the restricted distribution of dyed fibers collectively demonstrate that the microfibers adhering to the tools primarily derive from intentional fiber-working activities carried out at the site.

In summary, most microfibers observed on these tools are of anthropogenic origin and contemporaneous with the site’s occupation period. They are either associated with tool functions or derived from human activities within or near the site. Therefore, we propose that a substantial abundance of microfibers on tools, along with usewear patterns indicative of fiber processing on specific tools, would greatly strengthen confidence in attributing the fibers’ origins to tool functions.

Given the perishable nature of fiber materials, the airborne behavior of microfibers, the complexity of identifying microremains from different fiber types, and the multifunctional character of most Paleolithic tools, it remains highly challenging to recognize implements associated with fiber production in Paleolithic contexts. This paper, as a pilot project employing a multidisciplinary approach, seeks to address and overcome these challenges.

Our working hypothesis is that certain tools from Shizitan were used in fiber production. This hypothesis would be supported by a positive correlation among three lines of evidence: (1) the tools exhibit morphological or technological features consistent with specific stages of the fiber-production sequence; (2) usewear patterns indicative of fiber processing; and (3) the presence of abundant microfibers and associated elements on the tool surfaces.

## 4. Results

### 4.1 SZT Fiber types

**Fig 5:**
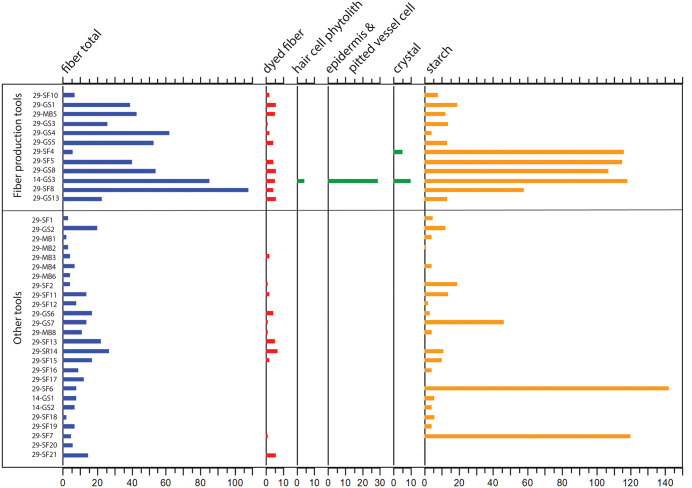
Comparison of multiple types of microbotanical remains (in quantity) between fiber production tools and other tools analyzed.

Following this working hypothesis, our analysis of residues and usewear traces on 38 stone tools from Shizitan (SZT) revealed consistent patterns indicative of fiber production on 12 artifacts (31.6%), including one microblade, four flake tools, and seven grinding stones. These tools are characterized by: (1) tool types commonly associated with fiber production for cutting, pounding, and scraping (SI, S4 Table); (2) usewear traces showing various levels of polish and/or fine striations, indicative of processing soft, silicious plant materials (SI, S4 Table); and (3) a significant presence of microfibers (n = 546; ranging 6–108; average 45.5 per tool) and fibrous plant elements (epidermis, vessel cells, crystals, and hair cells) (SI, S6 Table). In contrast, the remaining 26 tools showed less evidence of silicious plant processing in their usewear patterns and contained lower quantities of fibers (n = 241; ranging 0–27; average 9.3) (SI, S7 Table). A comparison of the microfossil remains between these two tool groups is illustrated in Fig 5, showing that while most tools were likely used for food processing (as indicated by presence of starch grains), the 12 analyzed tools contained notably high quantities of microfibers, associated dyed fibers, and fibrous plant elements. In the following discussion, we primarily focus on the fiber remains recovered from the 12 tools that display strong evidence of fiber production. It is important to note, however, that these tools were likely multifunctional—used for fiber processing and food procurement—rather than being specialized implements dedicated solely to processing a specific fiber type.

**Fig 6:**
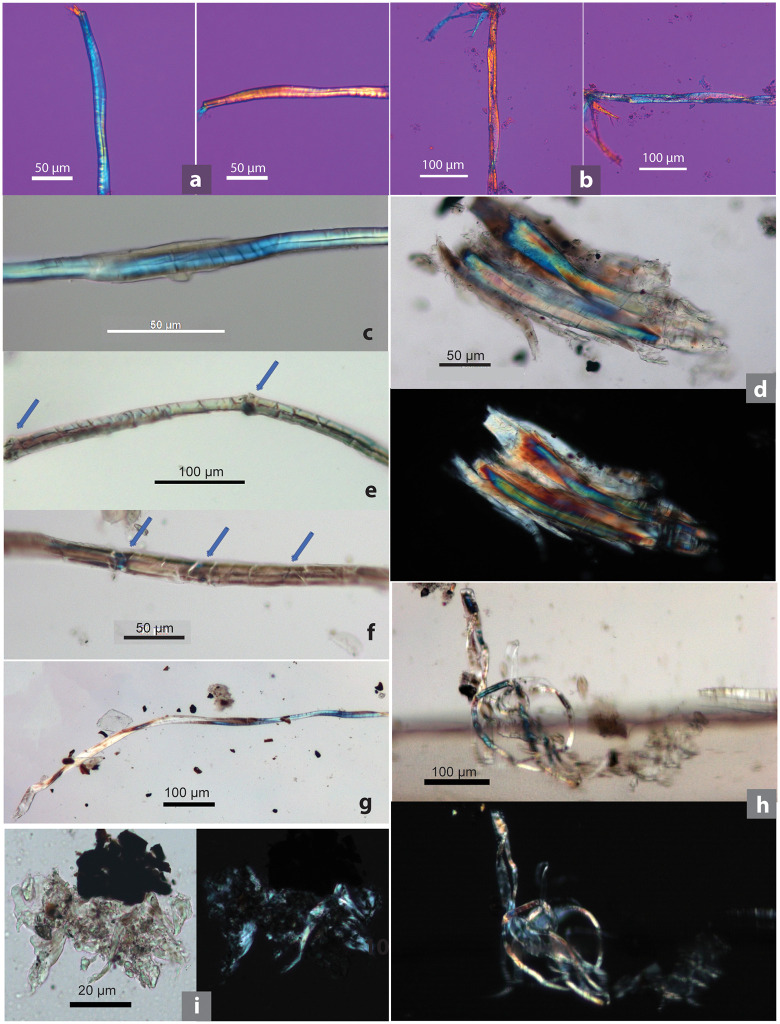
Microfibers uncovered from SZT lithic tools. **(a)** Z-twist type in fibrillar orientation; **(b)** S-twist type in fibrillar orientation; **(c)** bast fiber showing visible Z-twist fibrillar orientation; **(d)** bast fiber bundle; **(e)** bast fiber showing prominent dislocations (indicated by arrows); **(f)** bast fiber showing less prominent dislocations (indicated by arrows) and cross markings; **(g)** damaged fiber showing twisted ribbon form, likely hemp; **(h)** long and entangled fiber; **(i)** very damaged UNID fibers (d, i, j showing both brightfield and polarized views).

Of the 546 fibers recorded, 470 were identified as bast fibers, including 445 single fibers and 25 fiber bundles, accounting for 86% of the total fibers. The remaining 77 fibers (14%) were too damaged to determine taxonomy and were classified as unidentifiable (UNID). These unidentified fibers do not exhibit characteristics typical of cotton, wool, or synthetics (Fig 6; SI, S6 Table).

Due to the damaged condition of most fibers, only a limited number (n = 92) could be examined for fibrillar orientation. Among these, 87 exhibited Z-twist (15.9% of the total; 100% ubiquity), while five showed S-twist (0.9% of the total; 33.3% ubiquity), yielding a Z:S ratio of 17.4:1 (Fig 6a and 6b; SI, S6 Table). Z-twist fibers are characteristic of hemp, whereas S-twist are typically associated with flax.

Many damaged bast microfibers exhibit a twisted ribbon form (n = 72; 13.2% of the total; 100% ubiquity). This pattern is indicative of both hemp and flax fibers (Fig 6g and 6h; SI, S6 Table).

The cross-section diameters of bast fibers from SZT29 measure within the range of 5.12–44.11 µm, with an average of 17.51 µm (n = 482). This range largely overlaps with, but is slightly greater than, that observed for hemp and flax, likely reflecting damage to the archaeological fibers (Table 1).

**Fig 7:**
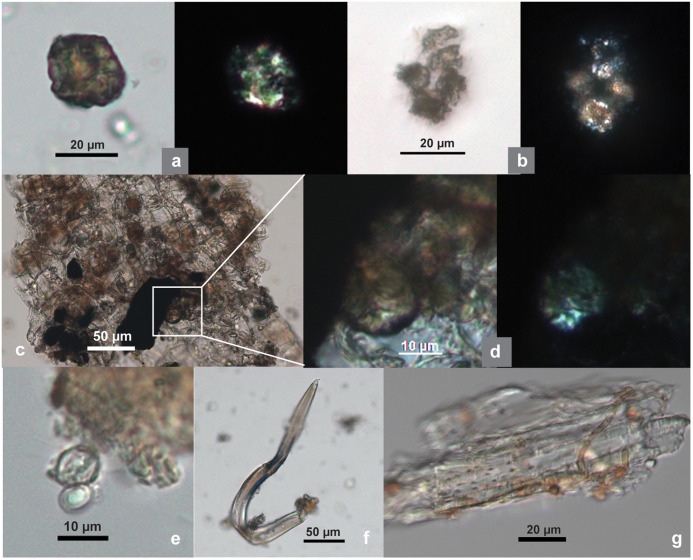
Bast fiber associated elements from SZT lithic tools. **(a)** A large clustered crystal, resembling velvetleaf (14-GS3); **(b)** a group of small clustered crystals, resembling hemp (29-SF4); **(c)** small clustered crystals attached to epidermis; **(d)** enlarged image of a crystal from (c); **(e)** yeast cells in budding process; **(f)** curved hair cell, resembling hemp; **(g)** pitted vessel cells. (Crystals, hair cell, and pitted vessel cells are comparable with corresponding elements from modern examples in Fig 2e–2g; all images from 14-GS3 except b).

The morphology of calcium oxalate crystals—occurring in clustered or solitary forms—has been used as a diagnostic feature for identifying fiber types [92]. Fifteen clustered-type calcium oxalate crystals, with size range of 5.11–26.86 µm, were identified from two tools. Their form and size range are consistent with those from hemp (Table 2). A flake tool, 29-SF4, yielding a group of five crystals, also exhibits very high polish on its edge (Fig 3b). A slab 14-GS3 has ten crystals, some of which were still attached to epidermal cells (Fig 7a–7d, compare with Fig 2g; S6 Table).

Four curved hair cell phytoliths were found on a slab (14-GS3) and resemble those from hemp (Fig 7f, compared with Fig 2e). Pitted vessel cells were also present in the 14-GS3 sample, most resembling those from hemp in morphology (Fig 7g, compare with Fig 2f).

Four yeast cells, appearing attached to an epidermis fragment, were identified on slab 14-GS3. They are round or oval, with a size range of 6.99–10.57 µm. Two cells appear to be in the budding process, indicated by the presence of small protrusions on the mother cells (Fig 7e). During retting processes, various microorganisms, including bacteria, molds, and yeasts (and the pectinolytic enzymes they produce), play a role in breaking down pectin and other substances binding the fibers to other plant material [95]. The presence of yeast on this slab might be the result of pounding retted fibers on the tool.

Based on these observations, we conclude that the bast fibers found at SZT14 and SZT29 may have originated from hemp and flax. Although it is difficult to estimate the percentage of each fiber type, hemp is likely the predominant one, supported by the higher frequencies of the Z-twist type than the S-twist type, the prevalence of twisted ribbon forms, the presence of clustered crystals and curved hair cells, and of pitted vessel cells consistent with those from hemp.

### 4.2 Fiber production process

Detailed analyses of usewear and microfossil remains on the 12 lithic tools suggest that fiber production was ongoing at SZT throughout Phases 1–4. The morphologies of usewear—such as varying degrees of polish, different forms of striations, and micropitting—observed on the various lithic tool types in this project are based on our previously published experimental studies [46,47,50,96]. The identification of usewear patterns and their associations with specific processing materials, as established through experimental studies, are documented in two published works [42,46] and summarized here. Based on the functional analysis of these tools, the production processes can be reconstructed as follows:

(1) Cutting fibrous plants: One microblade (29-MB5) and four flake tools (29-SF4, 5, 8, 10) were likely used as sickles or knives in similar activities. They exhibit high polish and fine, parallel and/or multi-directional striations along the cutting edge, suggesting use for cutting plants with relatively high silica content (S5 Table). Additionally, these tools reveal a relatively high quantity of microfibers (n = 6–108, with an average of 45.5) (S6 Tables). Microblades can be used in composite tools, with a row of blades inserted into a handle to form a long working edge, but the organic handles are rarely found archaeologically. One comparable example from North China is a composite microblade knife excavated from Donghulin near Beijing, dating ca. 10,000 cal BP (Fig 1C: 7) [31].
(2) Retting stems: The two SZT localities were situated near a river, which may have provided suitable water sources for retting. The presence of yeast cells attached to an epidermis fragment on 14-GS3 further supports a connection to the retting process (S6 Table; Fig 7e).
(3) Pounding fibers: Seven sandstone slabs (14-GS3; 29-GS1, 3–5, 8, 13) exhibit low to medium polish, consistent with processing soft plants (S5 Table), and show high quantities of microfibers (n = 23–85, with an average of 48.9) (S6 and S7 Tables). However, the polish on these slabs is notably lower than that observed on the microblade and flake tools. This difference is likely because the slabs served as supporting platforms while wooden pestles or handstones performed the pounding action, resulting in much less direct abrasion on the slabs compared to the cutting tools, and thus lower levels of polish.

Among the seven slabs, 14-GS3 shows the most diverse array of microfossil remains associated with fiber pounding. These include not only microfibers and microfiber bundles but also various related elements such as crystals, hair cell phytoliths, epidermis fragments, pitted vessel cells, and yeast cells likely linked to the retting process (Figs 5, Fig 7a and 7c–7g; S6 Table).

(4) Scraping fibers: Four flakes (29-SF4, 5, 8, 10), identified above as cutting tools, may also have been used as scrapers to remove impurities for producing more-refined fibers. This is suggested by the presence of high polish, fine striations perpendicular (transverse) and/or multi-directional to the edge, along with high quantities of microfibers and/or associated elements, such as crystals (Fig 5; S6 Table).
(5) In our experimental study on hemp fiber ribbon scraping, the residues on the scraper after use consisted of abundant fibers, epidermal fragments, and crystals (see Section 3.3), forming an assemblage that closely resembles the residue components found in these SZT samples (Figs 3j–3m, 5 and 7).

In summary, the multiple lines of evidence derived from usewear and microfossil analyses enable us to propose a hypothetical operational sequence, or *chaîne opératoire*, of fiber production—from the harvesting of fibrous plants to the manufacture of fine fiber products (see more in Discussion).

### 4.3 Dyed and pigmented fibers

Forty-six colored fibers were identified in 11 samples, representing 8.4% of the total fibers analyzed, with a ubiquity of 91.7%. Two types of coloration were identified: dyed and pigmented. Dyes are soluble in liquid media, allowing them to migrate into the fibers. In contrast, pigments are insoluble, and so they are typically used in conjunction with a binder, such as animal fat or albumin, which helps adhere them to the surface of the fibers [97].

**Fig 8:**
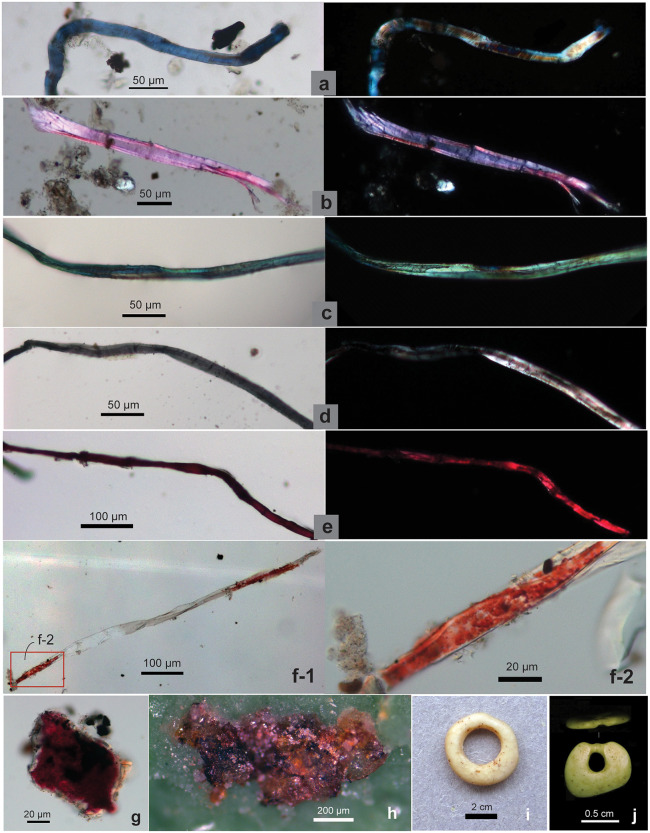
Dyed and pigmented fibers, hematite powder, and ostrich eggshell beads from SZT. **(a–e)** Fibers dyed blue, pink, green, black, and red; each fiber image showing in brightfield (left) and polarized (right) views (from SZT29); **(f)** red hematite pigment attached to a fiber, f-2 is an enlarged view of the left end of f-1; **(g)** hematite pigment attached to an unknown object; **(h)** hematite powder on PVS sample (f–h from 29-GS3); **(i)** ostrich eggshell bead showing red pigment on surface and perforation (SZT29 Layer 7 Top); **(j)** bead exhibiting wear traces (from SZT24).

When observed under a light microscope, undyed natural bast fibers typically appear polychromatic in both brightfield and polarized views, with the colors of the fibers not necessarily matching between these two microscopic views (Fig 2b; Fig 6d and 6h). Conversely, dyed fibers exhibit a relatively consistent color appearance under both brightfield and polarized light, matching the hues observed on the actual fiber materials, indicating that color expression is stable across optical conditions and can be reliably compared. This characteristic is demonstrated by bast fiber materials traditionally dyed black, blue, pink, red, and green in Yunnan (Fig 2h, 2i and 2k–2m for modern fibers; Fig 8a–8e for SZT specimens).

Differing from dyed fibers, pigmented fibers display a layer of pigment attached to the surface of the fiber (Fig 8f).

The dyed fibers from SZT display a range of colors, including blue (n = 21; 3.8% of total; 83.3% ubiquity), pink (n = 11; 2.0% of total; 75.0% ubiquity), black or grey (n = 11; 2.0% of total; 50.0% ubiquity), green (n = 1; 0.2% of total; 8.3% ubiquity), and red (n = 1; 0.2% of total; 8.3% ubiquity) (S6 Table; Fig 8). The observed color range is broader than that of previous findings from sediment NPP samples, which reported pink, blue, black, and grey [11].

A fiber recovered from a grinding slab (29-GS3) exhibited red pigment on the surface of its ends, while the middle section of the fiber, showing a twisted ribbon form, appeared to be damaged and devoid of pigment (Fig 8: f-1 and f-2). The morphology of this fiber resembled that of damaged hemp or flax. Additionally, several small unidentifiable objects from the same tool also exhibited the presence of red pigment (Fig 8g). PVS peels taken from two slabs (29-GS3, 29-GS5) and one handstone (29-GS7) revealed clusters of red mineral powder (Fig 8h). In our early study of these tools, portable X-ray fluorescence spectrometry (pXRF) analysis of some of these powder remains confirmed the presence of a significant iron component, likely originating from hematite. Additionally, usewear traces on these grinding stones displayed deep striations and fractures, indicative of their potential use for processing hematite [42] (S5 Table).

## 5. Discussion

The examination of fiber remnants and usewear patterns on SZT tools provides both direct and circumstantial evidence supporting the production of bast fiber. The activities related to fiber production contribute to a broader and more nuanced comprehension of the adaptation strategies employed by Upper Paleolithic communities in this region, especially in response to LGM climatic change. These perspectives are summarized as follows.

### 5.1 Reconstruction of the fiber production *chaîne opératoire*

Our proposed working hypothesis is supported by the observation that 12 tools exhibit both a high concentration of bast microfibers along with fibrous plant elements (Fig 5), and usewear traces, characterized by high polish and fine striations consistent with fiber processing, as seen in our fiber processing experiments. Specific tool forms—including flakes, microblades, and slabs—appear to facilitate distinct stages in the operational sequence of fiber production: cutting fibrous plants, pounding fiber ribbons, and scraping fiber ribbons. Additionally, the presence of yeast cells on a slab likely used for pounding retted fiber ribbons suggests involvement in the retting process. All these procedures have been well documented ethnographically [81,82,85] (SI, S8 Table). Therefore, we argue that these interrelated lines of evidence constitute direct archaeological evidence for fiber production.

As discussed earlier, pounding fiber ribbons and scraping off impurities are essential procedures in the traditional production of bast fiber textiles but are not necessary for processing fibers to be used in cordage, as seen ethnographically (S8 Table). These two processes were also present at the Neolithic Peiligang and Shimao sites in northern China, as evidenced by pounding tools and scrapers associated with fiber and textile remains [13,22,23], as discussed in Section 1. These findings align with studies of traditional hemp processing methods in Asia and Europe by Clarke [98], indicating that softening fiber ribbons by pounding was a common practice in both regions. However, based on this study, while fine fiber extraction for textile production in East Asia has typically been achieved through scraping fiber ribbons, in Europe, this process has been carried out through scutching and hackling. Consequently, the identification of tools at SZT with evidence of likely having been used in pounding and scraping bast fibers raises questions about why such fiber processing techniques—which are associated with traditional bast fiber textile production rather than making cordage in East Asia—would be present at SZT. This issue will be further discussed in Section 5.6 below.

The bast fibers identified on SZT tools may have predominantly consisted of hemp, accompanied by a smaller proportion of flax. Reasons for this potential preference for hemp over flax should be further explored, but one possibility is due to a preference for hemp’s longer fibers, as hemp plants typically grow 1–3 meters tall [54:75], whereas wild flax (*Linum stelleroldes*) in the Shanxi region only reaches heights of 40–70 cm [68:3], although cultivated flax can grow taller (S1D–S1F Fig).

### 5.2 Pigmented fiber

Two grinding stones (slab 29-GS3, handstone 29-GS7; Fig 1D: 9 and 11) had traces of what is likely hematite powder [42]. The slab also shows a fiber with hematite pigment on it (S6 Table). We would argue that deliberate pigmentation of this fiber is highly probable, as hematite powder does not naturally adhere to fibers; it typically requires an artificially-applied binder [97]. If this is the case, this could stand as direct evidence of a purposeful fiber production technology involving hematite pigment. The processing of hematite ores using grinding stones as well as the application of the pigment to various other materials have been documented at multiple Upper Paleolithic sites in the Shanxi region, including Shizitan and Xiachuan [24,47,99]. Hematite pigment has also been identified in the coloring of beads from SZT29 [72] and in the creation of painted rock art images near SZT1 [24]. Shanxi is renowned for its plentiful and extensively scattered iron deposits, encompassing hematitic ores. One such iron deposit is situated approximately 60 km east of SZT [100:231–233].

Considering these collective findings, the processing of hematite pigment appears to have been a common activity among Upper Paleolithic communities in the southern Shanxi region. Our observations support the possibility that fibers were one of the materials subjected to this coloring technique.

### 5.3 Dyed fibers

Approximately 8.4% of the fibers that we observed from SZT (46/546) display evidence of dyeing, with the prevalent colors including blue, pink, and black/grey, and with a smaller number of fibers exhibiting green or red hues. Natural dyes utilized for coloring fibers typically comprise soluble organic compounds that can be extracted from plants (roots, leaves, flowers, bark/branches, fruits/seeds), animals (insects), or minerals [97]; fungi and bacteria can also be used in dying [101]. The earliest potentially dyed bast microfibers were discovered in 30,000-year-old deposits at Dzudzuana Cave in Georgia [10], and evidence for processing of woad (*Isatis tinctoria*) leaves on grinding stones, possibly for making blue dye, has also been identified at Dzudzuana Cave, dating to 34−32,000 years ago [9]. Dyed microfibers (black) have been found on the Upper Paleolithic tools at Peiligang in Henan (ca. 26,000 cal BP) [13]. Therefore, the presence of purposeful dying of fibers at SZT would not be unprecedented as an Upper Paleolithic technology.

In China, various plant-based materials have been used for dye production since antiquity [summarized and references within: [102–104]]. The root of madder (*Rubia cordifolia,* known as *qiancao* in Chinese) was utilized to create a range of reddish dyes, including yellow, orange, pink, and red [105]. Several plant species were employed for producing blue dye, among which Chinese woad (*Isatis indigotica*, known as *songlan* in Chinese) is the most widely distributed in North China [106,107]. Acorn caps from *Quercus* oak (*zaodou* in Chinese) could produce black dye [104]. The tender fruit and stem bark of Dahurian buckthorn (*shuli* in Chinese, *Rhamnus davurica*) could yield green dye [108]. Traditional dying techniques are still practiced in some regions today. In the SZT area, local people utilize goosefoot (*huihuicai* in Chinese, *Chenopodium album*) for grey dye and green walnut peels (*Juglans* sp.) for green dye.

In more remote areas, indigenous ethnic groups employ a variety of plant species, with various parts of the plants, including flowers, bark, stems, tubers, and roots, producing dyes for coloring their customary clothing. For example, the Bai people in Yunnan, southwest China, collect walnut peels to make black dye [109].

Coloring fibers with plant dyes is a complex and time-consuming process that involves various dye extraction methods, multiple stages of dyeing, and the incorporation of additional mordants (such as plant ash to enhance darker color), depending on the dye plant used [104:78–88]. In addition, bast fibers are also challenging to dye due to the lack of bonding between natural dyes and the fiber. Therefore, pretreatment through a mordanting process is necessary [110]. This highlights the inherent difficulties involved in dyeing fibers. The microfiber remains from SZT exhibit a high number of dyed fibers in a wide range of colors, requiring intricate processes with additional chemicals. Therefore, it is unlikely that these fibers were accidentally dyed by natural processes.

For example, Chinese woad *Isatis indigotica* (belongs to the family of Brassicaceae or Cruciferae) is a cold-tolerant and drought-tolerant plant well adapted to temperate environments [111]. Blue dye extraction from its leaves traditionally involves harvesting fresh leaves during the summer to early autumn growing season (June–October) before the onset of frost, steeping or fermenting them in warm water to hydrolyze indole precursors (e.g., indican) into solution, then increasing the alkalinity—such as by adding plant ash—and aerating the liquid to oxidize leuco-indigo into insoluble indigotin, which precipitates and can be collected as a blue pigment. The subsequent dyeing step entails immersing fibers in the reduced dye bath, followed by exposure to air to oxidize and fix the blue color on the material [67: 182, 104: 78-79, 112]. Given its ecological tolerance and the relative simplicity of this extraction process, even rudimentary technological knowledge among Upper Paleolithic groups could have enabled the use of locally available indigo-type dyes. Therefore, we propose that SZT people may have intentionally used dye-making plants available in the region.

Madder, Chinese woad, *Quercus* oak, Dahurian buckthorn, goosefoot, and walnut all grow in Shanxi [68,113,114] and can be found in today’s Jixian and the surrounding counties. Pollen studies conducted on sediment samples from the SZT site indicate the presence of Cruciferae plants during the early Holocene, while *Quercus* existed in the pre-LGM and post-LGM periods. Chenopodiaceae occurred throughout the pre-LGM, LGM, and post-LGM periods in the region [11,34]. The various colorations on fibers also partially align with the availability of local dye-producing plants both in the past and present (S9 Table). Thus, the Paleolithic inhabitants of SZT may have potentially sourced some of these plants from the nearby area to produce and dye fiber materials on-site.

Furthermore, if these colored fibers at SZT are intentionally colored, this would indicate a deliberate modification of the fiber materials, and some consideration should be given to potential reasons for doing this. While the specific purposes of such coloring cannot be determined from the available archaeological evidence, color treatment in fiber technologies in later periods is sometimes associated with aesthetic, functional, or symbolic uses [2,5,115].

### 5.4 Needles, beads, and pendants as indirect evidence of fiber production

Excavations conducted at several SZT localities have yielded an eyed bone needle and numerous beads and pendants made from ostrich eggshell, clamshell, and bone, dating to 27,000–11,350 cal BP [71,72,116]. The existence of the eyed needle suggests the possibility of sewing and stitching [38,117,118]. While there is no direct evidence of the specific materials used with the needles, plant fibers could be used for the stitching thread or in the materials that would have been sewn together (cloth textile, for example), although using animal products (skins, sinew, hair fibers) would also be possible. Some beads and pendants exhibit significant wear traces, suggesting prolonged use through the repeated action of stringing and tying with cordage, and from wearing the strung objects (Fig 8j). Some ostrich eggshell beads exhibit pigmentation on their perforations and surfaces that fall in the color range of the fiber pigmented using hematite (Fig 8f and 8i) [71,72,116]. Notably, ostrich eggshell beads and hematite have also been found at the Upper Paleolithic deposits at Peiligang in Henan, about 500 km south of SZT [13], suggesting a widespread practice.

These findings, consistent with similar materials discovered at contemporary sites elsewhere in the world [37], provide indirect evidence for the use of thread, string, or rope, suggesting that the fiber products produced at SZT likely included cordage employed for diverse purposes.

### 5.5 Seasonality of fiber production coinciding with other activities

Traditionally, fiber production has been closely interconnected with food procurement activities in sedentary agricultural societies, as illustrated in the ancient Chinese poem “Qiyue” in the “Binfeng” chapter (The Seventh Month in Airs of Bin) of the *Classic of Poetry* (11^th^ – 7^th^ centuries BC) [119]. However, Paleolithic foragers’ activity cycles are poorly understood. By comparing the timelines of these two types of activities, we can potentially enhance our comprehension of Paleolithic site function and adaptive strategies at SZT29. To understand the seasonal scheduling of traditional fiber production, we consulted historical accounts and ethnographic records from China, which are detailed below. Assembling these individual accounts together enables the reconstruction of a more comprehensive and coherent understanding of the timing of traditional fiber production. The study of seasonal scheduling for fiber production based on Holocene data is not intended to reconstruct its absolute temporal framework during the LGM. However, our data also includes both pre-LGM and post-LGM contexts, indicating the continuation of fiber production throughout the entire occupational sequence of the site (S5 and S6 Tables). This continuity suggests that certain plants may have survived climatic fluctuations in the SZT area. Our discussion therefore aims to explore whether fiber production and food procurement could have been concurrent activities at SZT, assuming that these plants were locally collected, even though their growing seasons were shortened during the LGM.

**Fig 9:**
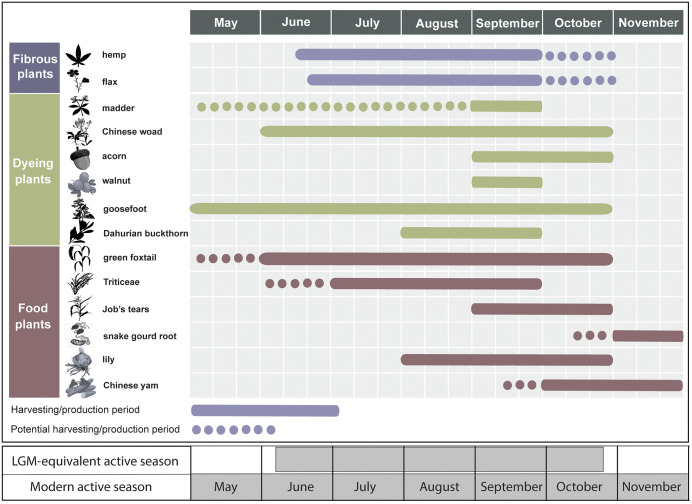
Estimated seasonal activities of fibrous, dyeing, and food plants based on Holocene data and LGM-equivalent reconstructions. **Upper:** Overlapping ranges of harvesting and production periods for the three plant categories occurring between June and October. **Lower:** Comparison of modern (May–November) and estimated LGM-equivalent (early June–late October) active seasons, calculated using a −7 °C temperature offset for Shanxi (shaded areas).

The entire process of bast fiber production, including harvesting, retting, decortication, pounding, scraping, splitting and splicing, thread-making, dyeing, and potentially weaving (S8 Table), is labor-intensive and highly seasonal. To obtain the best-quality fibers, hemp is typically harvested when the staminate plants have finished flowering but before the seeds have ripened [120]. In East Asia, hemp harvesting generally begins in mid-June [84]. An ancient Chinese agricultural manual from the 1^st^ century BC, the *Fan Shengzhi Shu*, records that the optimal time for retting hemp is 20 days after the summer solstice, which would thus be in early July [121]. The poem “Qiyue” in the *Classic of Poetry* mentions that the splitting, splicing, and twisting of hemp thread takes place in the eighth month of the lunar calendar (*bayue zaiji*), which corresponds to September of the solar calendar [119]. Consequently, the fiber production process would have taken several months, likely spanning from mid-June to September and possibly beyond (Fig 9; S10 Table).

Different dye-producing plants require specific collection seasons and processing methods. As summarized by Chen [104], Chinese woad leaves are typically collected from June to October. They are then pounded into pieces and fermented in water for several days before being used for dyeing fiber materials [112]. Madder roots, on the other hand, can be collected from May to September, with autumn-harvested roots considered to be of better quality. These roots’ dyes require the application of a mordant, such as plant ash, to bind the dyes to the fibers. Madder can produce various colors, including yellow, orange, and different shades of red, with deeper shades of red achieved by dyeing the fibers multiple times [104: 78–81]. Based on the various colors present on the fibers from SZT, we estimate that the collection and processing of dye plants to obtain these colors primarily occurred from June to September, which overlaps with the time frame for bast fiber production (Fig 9; S10 Table).

Previous starch granule analysis revealed that SZT stone tools were utilized for processing a variety of food plants, including wild millet, Job’s tears, Triticeae grasses, yam, lily, and snake gourd roots [42,46]. These plants reach maturity for harvesting at different times, spanning from May to November, with most from June to October. This period coincides with the time ranges for fiber and dye production (Fig 9; S10 Table).

Notably, these Holocene-based timeframes must have been shorter during the LGM, when temperatures in the Shanxi region are estimated to have been 6.7–7.4 °C lower than at present [33]. Based on this temperature difference, the reconstructed LGM seasonal growing pattern suggests that the modern active period of approximately May–November would have been compressed to roughly early June–late October (Fig 9; see SI Section 3). Such a shortened and delayed warm season would have constrained the availability of wild plant resources and limited the timing of activities related to fiber processing and other temperature-dependent crafts. The calculation method used for this reconstruction is presented as a heuristic approach, intended to illustrate general seasonal trends rather than to provide a precise climatic model.

Nevertheless, the overlapping seasonality of locally available plant types implies that food and fiber resources may still have attracted people to the site during the summer and early autumn. Activities undertaken during this period would have included food procurement, the harvesting and processing of plant fibers, and possibly social gatherings, exchange, and ritual events.

### 5.6 Intensification in fiber production amid climatic change

In SZT29, the earliest fiber remains were found in Layer 8, indicating the existence of fiber technology predating the LGM. However, there is a notable increase in fiber production intensity, marked by higher quantities of microfiber remains, starting during Initial LGM (26,000–24,000 cal BP) and persisting into the Late Glacial period (S6 Table). This shift coincided with several unprecedented developments in material culture, including the introduction of microblade pressure production technology, the use of beads, the making of bone needles, and the processing of hematite powder for pigmenting beads, fiber materials, and rock art, as well as the consumption of wild millet [11,42,43,72].

Additionally, the recognition of fiber processing at SZT necessitates a re-evaluation of assumptions about the functions of various tool types at Upper Paleolithic sites in North China. For instance, in other regions of Eurasia, Gilligan and colleagues [38, 39:66–79] suggest that blades and scrapers were primarily used for cutting and scraping hides to create clothing, particularly complex garments suited to cold climates. Functional studies of Upper Paleolithic grinding stones and knapped lithic artifacts worldwide have often emphasized their role in food processing [46,122–125], with limited attention given to non-food plant materials [9,87,88]. Our research proposes that microblades, flake scrapers, and grinding stones may have also been used in fiber production, particularly involving hemp and flax in East Asia. Furthermore, if grinding stones and scrapers were employed in pounding and scraping fibers for textile-making, it suggests that these techniques could date back to the Upper Paleolithic era in East Asia.

These developments also correlate with demographic shifts and likely new forms of social interactions deriving from the depopulating of more northerly regions during the LGM (e.g., the Shuidonggou site is abandoned) and migrations of surviving populations southward [35]. This is also tied to the still poorly understood advent of microblade pressure production, with the earliest known sites dating to ca. 28–27,000 cal BP, found in North China [11,126–128]. Since, generally speaking, sickles traditionally allow for the more efficient cutting of cereal and fiber plants [84], although no examples are known from Paleolithic China, we hypothesize, based on the SZT29 microblade usewear, that microblades may have been used in composite tools as sickles to cut wild millet and fibrous plants like hemp. Additionally, some SZT shell pendants were identified as from marine species originating from coastal areas, located at least 800 km to the east. This implies that people at SZT were likely engaged in extensive exchange networks for acquiring special decorative objects [71,72,116]. We also hypothesize that the presence of early (for North China) decorative objects at SZT such as ostrich eggshell and marine shell beads and pendants, the use of hematite pigments, and the presence of dyed fiber materials might also be linked to new forms of social signaling using material culture among hunter-gatherers in LGM North China that would be tied to new forms of social organization and information exchange [72,129]. These new forms of materiality (microblades, bast fiber processing, dyeing fibers, ostrich eggshell beads, and hematite) in the SZT area also find parallels with those at the contemporary Peiligang site in the Central Plains [13], suggesting that broader cultural networks extended beyond the Loess Plateau region.

We see these transformations that occur during this period of significant climatic fluctuations as key behavioral adaptations that underscore the new forms of human resilience emerging in this period as hunter-gatherers restructured the forms and extent of social interactions and exchange and came to more effectively and diversely utilize natural resources during the LGM.

## 6. Conclusion

In this study, the integrated analyses of tool types, usewear traces, and microfibers on the tools provide multiple lines of evidence for reconstructing bast fiber production dating to 28,000–18,000 cal BP in North China. These findings can also be correlated with patterns of natural resource availability and prevailing climatic conditions. While any of these lines of evidence considered individually could be seen as circumstantial, their combined and interrelated occurrence within the same assemblage strengthens the interpretation that they constitute direct archaeological evidence for fiber production in the SZT case.

Climate fluctuations during this period that encompasses the LGM also spurred population movements from more northerly regions to more southerly latitudes, including the southern Loess Plateau, where the SZT site cluster is located along with other Late Upper Paleolithic sites in the same general region (e.g., Xiachuan, Longwangchan), which appear to be among the most northerly inhabited sites during the LGM [130,131]. We correlate the challenging cold, dry conditions of the LGM with an apparent increase in, and the development of, fiber production (including microblade processing and the use of dyes and pigments). The SZT evidence indicates that Upper Paleolithic hunter-gatherers may have processed locally available hemp and flax to produce fiber products, likely cordages and potentially other materials.

Since bast fiber processing that includes the stages of pounding them for softness and removal of the epidermis and coarse fibers is ethnographically associated with textile production rather than cordage making (which does not include these two stages in China), and given that Neolithic tools used for textile manufacture included pounding tools and scrapers (both present at SZT, as well), we hypothesize that bast fiber production at SZT also may have been used in making textiles, as there is evidence present for these production stages at SZT that ethnographically are known to be reserved for fibers used in textiles and not in cordage. The presence of eyed needles would offer the possibility that such textiles would even have been sewn into plant-based clothing. Although no direct archaeological evidence of such textiles or clothing has been found, indirect evidence includes the presence of an eyed needle at SZT29 and dyed fibers in multiple colors. The presence of dyed fibers at SZT would have required the gathering and utilization of dyes extracted from plants to produce fiber products in the various colors identified in the samples. Coloration may have played symbolic roles and could have been used in social signaling, and the employment of hematite powder for pigmenting fibers, beads, and rock art at SZT29 and other Shizitan localities could also be related to such roles. These changes likely facilitated more active interregional interactions, ritual activities, and the development and maintenance of information exchange networks, which in turn further accelerated the production of ornaments and fiber-based materials associated with social signaling. These developments occurred concurrently with the intensification of broad-spectrum subsistence practices, including the earliest known utilization of wild millet as a food source [42].

SZT was not the sole area in the middle Yellow River region where Paleolithic populations engaged in fiber production, given that the Peiligang site also revealed evidence of fiber production during the Late Upper Paleolithic [13]. Fiber products were likely to have been instrumental in enhancing human resilience to the cold climatic conditions of the LGM, especially since the crafting of complex clothing (and multi-layered, fitted garments) for better thermal insulation and protection from the elements would have been essential for survival [38,39]. This proposition needs to be tested with more data in the future.

Finally, from a methodological perspective, this project employs a multi-faceted approach—particularly integrating plant microfossil and lithic usewear analyses—to demonstrate how fiber production can be investigated archaeologically even in the absence of preserved macrofiber remains. However, the available data and analytical methods do not allow us to explore other possible uses of these plants (e.g., medicinal or dietary). Similarly, evidence for the final manufacturing stages of fiber products, following pounding and scraping, is lacking, as these steps may not have involved stone tools or other more-commonly preserved artifacts. Our reconstruction therefore presents only a partial view of the operational sequence of fiber production.

## Supporting information

S1 Fig Landscapes of Shizitan Localities 14 and 29 and fibrous plants of northern China discussed in this study. (TIF)

S2 Fig Residue sampling and experimental food processing. (TIF)

S3 Fig SZT29 stone tools analysis. (TIF)

S4 Fig Structure of bast fiber and other fibers for comparison. (TIF)

S5 Fig Experimental fiber processing. (TIF)

S6 Fig Usewear traces on different stone materials after the processing of various bast fiber plants. (TIF)

S1 Table Radiocarbon dates by layer from Shizitan 29. (DOCX)

S2 Table Radiocarbon dates by layer from Shizitan 14. (DOCX)

S3 Table SZT 14 residue and usewear sampling record in the Shanxi Museum, Taiyuan, 2009–2010. (DOCX)

S4 Table Usewear record of SZT29 tools. (DOCX)

S5 Table Usewear traces on 12 SZT fiber production tools. (DOCX)

S6 Table Microfibers and other residue remains from the 12 analyzed SZT fiber production tools. (DOCX)

S7 Table Microfossil remains from the other 26 analyzed SZT tools. (DOCX)

S8 Table Ethnographic studies of the hemp production process. (DOCX)

S9 Table Colorations of dyed fibers and possibly related dyeing plants in the Shizitan area. (DOCX)

S10 Table Seasonality of plants related to fiber and food production available in Shanxi. (DOCX)
