# Supplementary figures and images for: Unveiling bast fiber production in Upper Paleolithic North China: Microfibers and usewear traces on stone tools from Shizitan
Source: PLoS One. 2026 Apr 13;21(4):e0346767. doi: 10.1371/journal.pone.0346767 (PMC13075717; doi:10.1371/journal.pone.0346767)

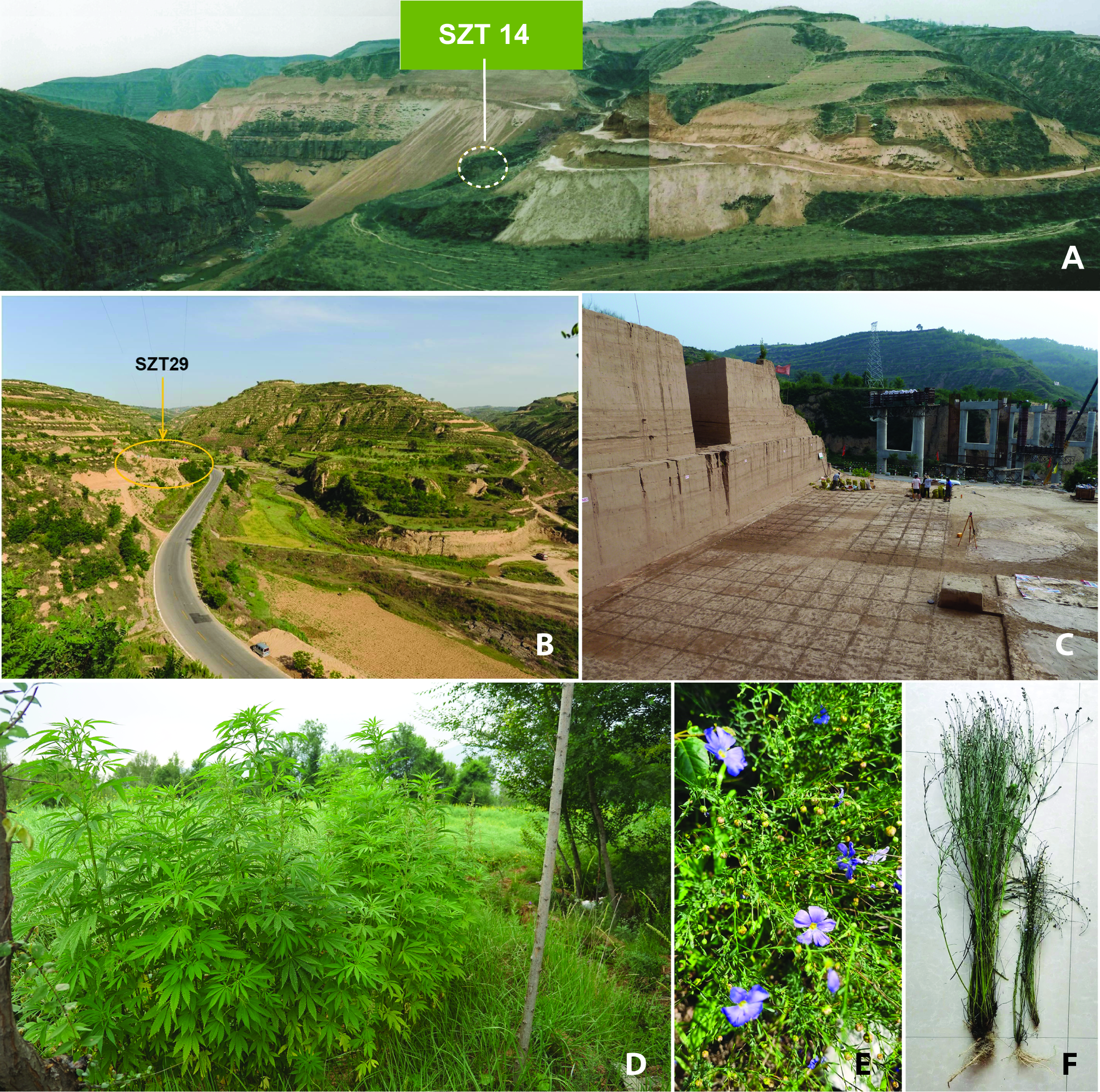

Supplement: S1 Fig — (TIF) [file pone.0346767.s001.tif]

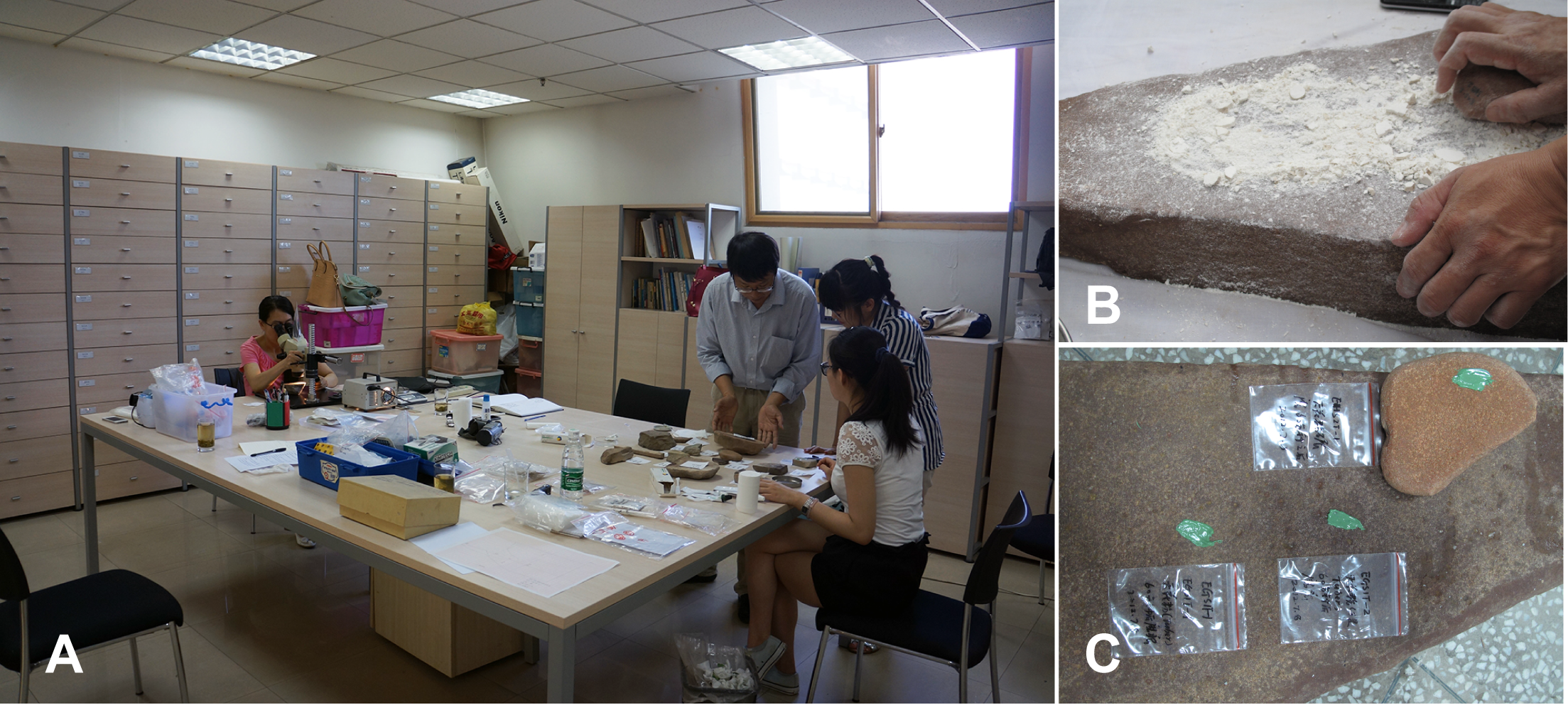

Supplement: S2 Fig — (TIF) [file pone.0346767.s002.tif]

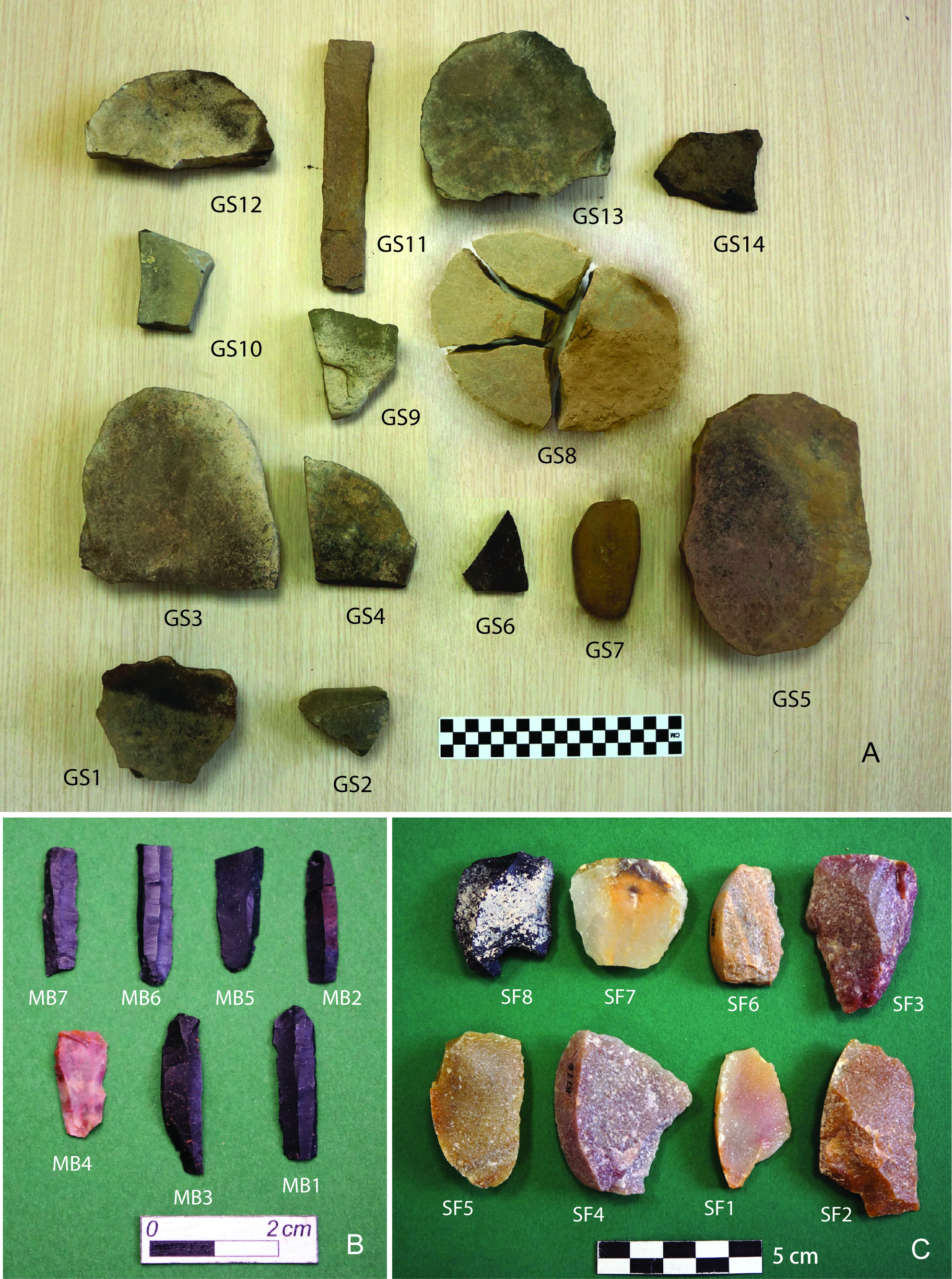

Supplement: S3 Fig — (TIF) [file pone.0346767.s003.tif]

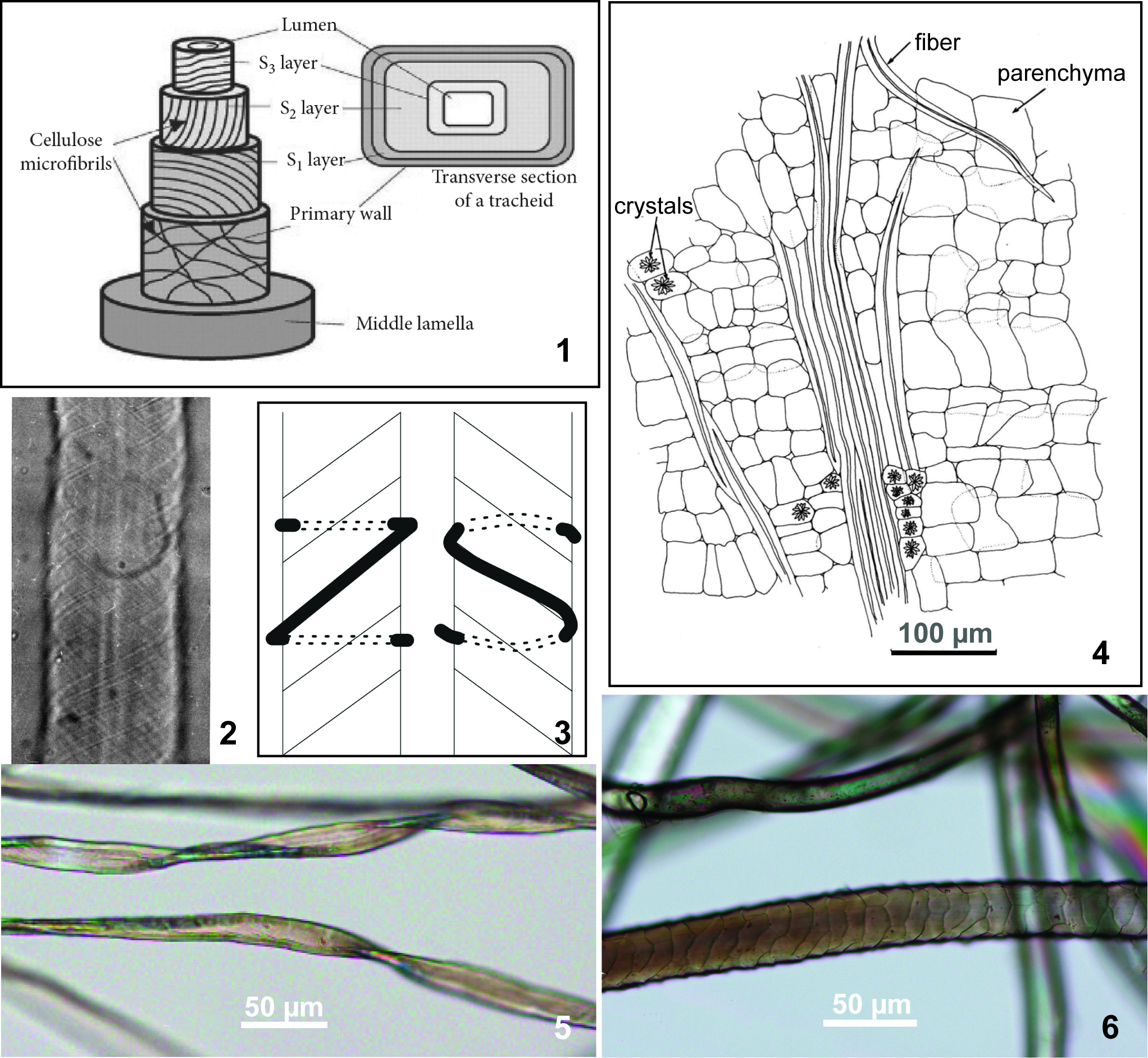

Supplement: S4 Fig — (TIF) [file pone.0346767.s004.tif]

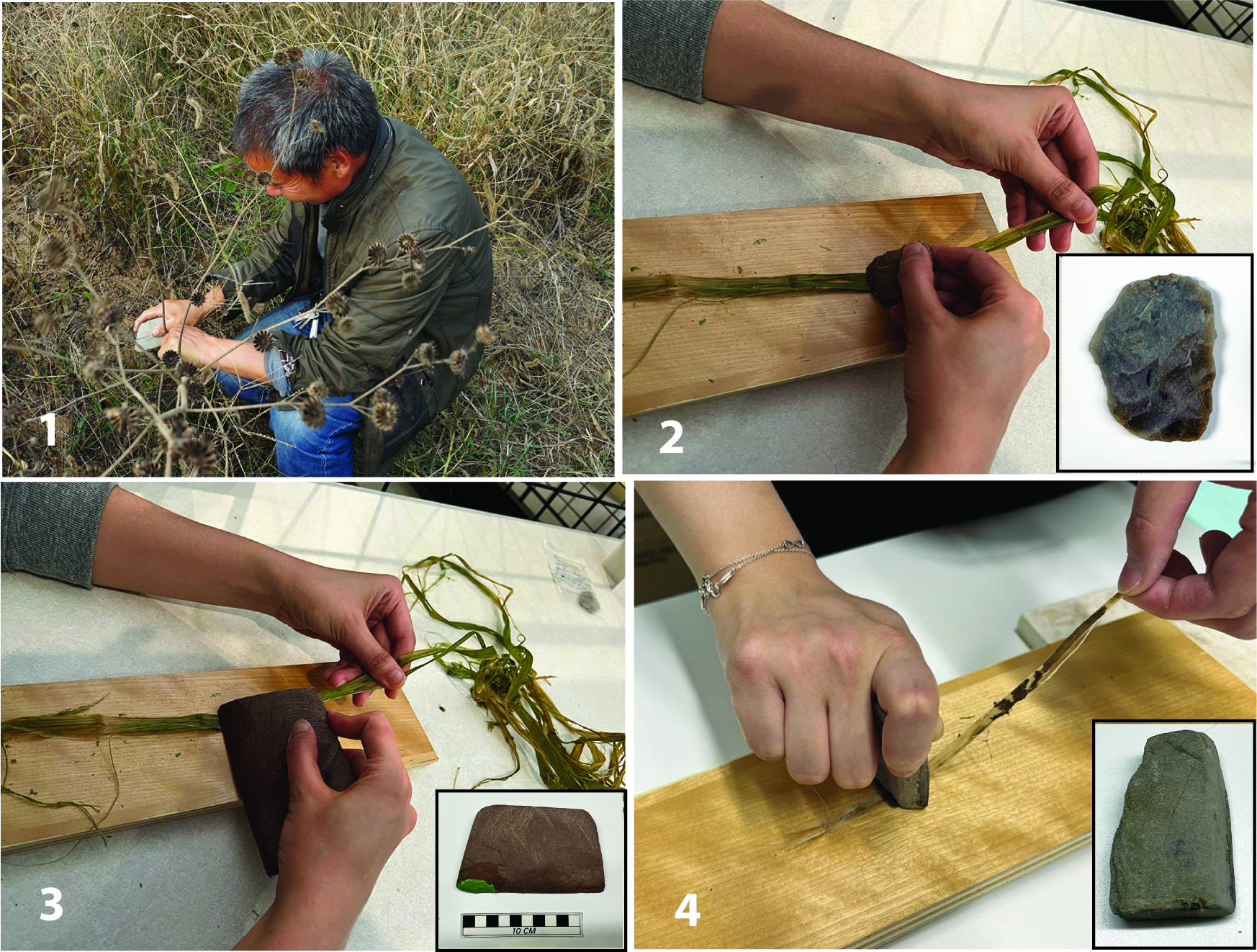

Supplement: S5 Fig — (TIF) [file pone.0346767.s005.tif]

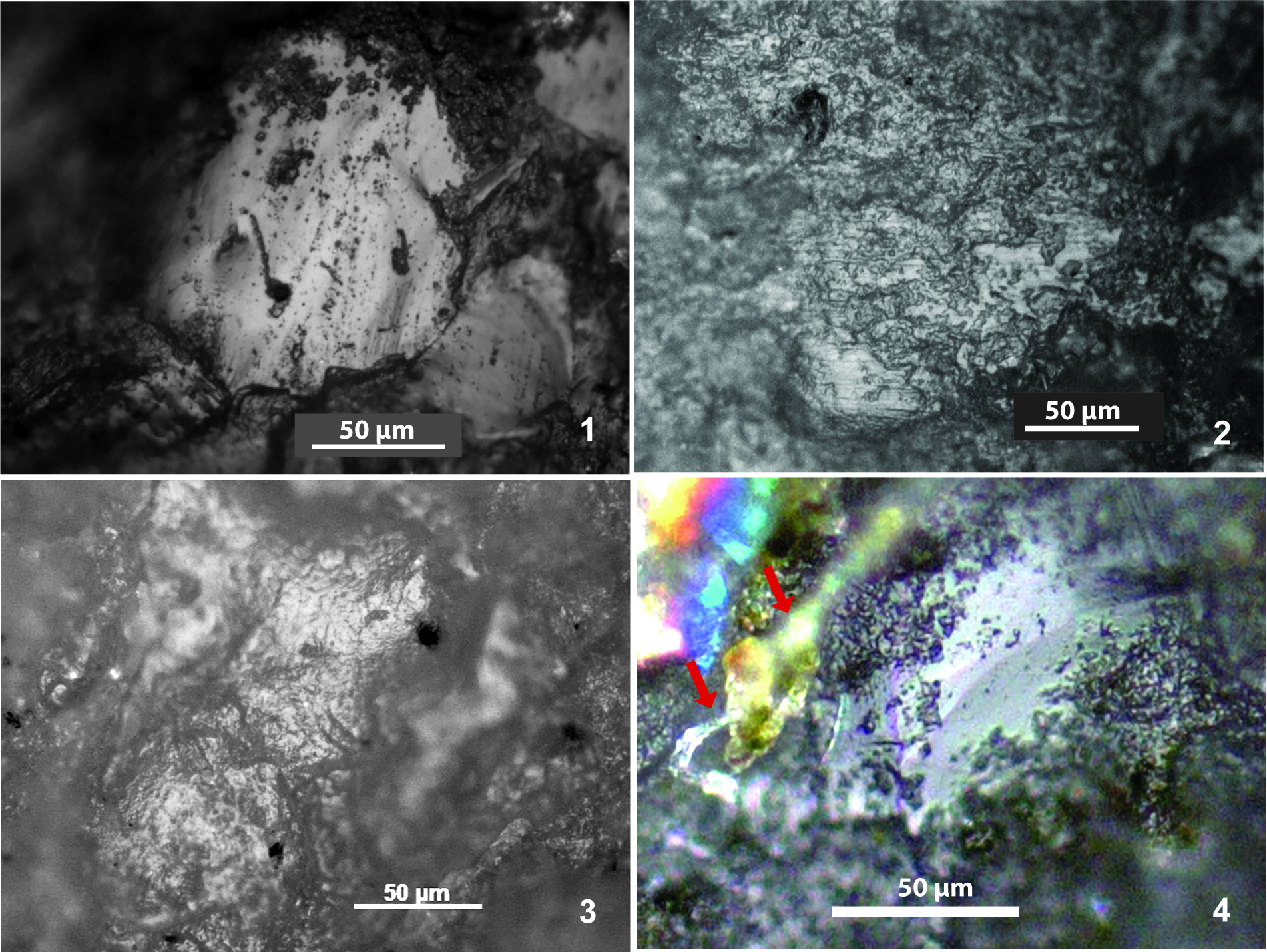

Supplement: S6 Fig — (TIF) [file pone.0346767.s006.tif]
