# Supplementary material for: Unveiling bast fiber production in Upper Paleolithic North China: Microfibers and usewear traces on stone tools from Shizitan
Source: PLoS One. 2026 Apr 13;21(4):e0346767. doi: 10.1371/journal.pone.0346767 (PMC13075717; doi:10.1371/journal.pone.0346767)
Supplement: S1 Table — (DOCX) [file pone.0346767.s007.docx]

**S1 Table Radiocarbon dates by layer from Shizitan 29 [28].**

|  | **Lab No.** | **Material** | **Layer** | **Depth (cm)**[**^a^**](#bookmark1) | **^14^C age (yr BP)**[**^b^**](#bookmark2) | **Calibrated age**[**^c^**](#bookmark3)  **(yr BP, 95.4%)** |
| --- | --- | --- | --- | --- | --- | --- |
| 1 | BA10129 | Bone | 1 | 63 | 11,175 ± 60 | 13,152-12852 |
| 3 | BA101412 | Bone | 1 | 63 | 11,390 ± 50 | 13,332-13106 |
| 4[^d^](#bookmark4) | BA101413 | Bone | 2 | 199 | 9190 ± 45 | 10,456-10245 |
| 5 | BA101414 | Bone | 2 | 203 | 14,650 ± 70 | 18,026-17623 |
| 6 | BA10132 | Bone | 2 | 200-206 | 15,725 ± 80 | 19,187-18793 |
| 7 | BA10131 | Charcoal | 2 | 248 | 16,760 ± 65 | 20,443-20007 |
| 8 | BA101416 | Bone | 2 | 276 | 15,390 ± 70 | 18,811-18502 |
| 9 | BA101419 | Bone | 3 | 282 | 17,200 ± 50 | 20,936-20564 |
| 10 | BA10133 | Bone | 3 | 303 | 17,360 ± 60 | 21,181-20711 |
| 11 | BA101420 | Bone | 4 | 465 | 17,500 ± 70 | 21,398-20885 |
| 12 | BA10134 | Bone | 4 | 469 | 16,170 ± 50 | 19,703-19316 |
| 13 | BA10135 | Bone | 4 | 605 | 16,930 ± 50 | 20,598-20217 |
| 15 | BA101422 | Bone | 4 | 620 | 16,750 ± 80 | 20,460-19979 |
| 16 | BA101421 | Bone | 4 | 622 | 18,570 ± 60 | 22,587-22312 |
| 17 | BA101423 | Bone | 4 | 624 | 19,210 ± 80 | 23,444-22892 |
| 18 | BA10136 | Tooth | 4 | 640 | 17,040 ± 60 | 20,753-20340 |
| 19 | BA10137 | Bone | 5 | 772 | 18,360 ± 70 | 22,426-21974 |
| 20 | BA10485 | Charcoal | 5 | 787 | 20,420 ± 80 | 24,922-24268 |
| 21[^d^](#bookmark4) | BA10486 | Bone | 5 | 787 | 11,980 ± 50 | 14,014-13722 |
| 22 | BA101426 | Bone | 5 | 750.5 | 19,650 ± 100 | 23,977-23381 |
| 23 | BA101427 | Charcoal | 5 | 751.5 | 19,510 ± 70 | 23,764-23193 |
| 24 | BA101428 | Charcoal | 5 | 750.5 | 19,940 ± 70 | 24,233-23760 |
| 25 | BA101429 | Charcoal | 5 | 804 | 19,710 ± 80 | 23,999-23484 |
| 26 | BA101430 | Charcoal | 5 | 801.8 | 19,860 ± 70 | 24,135-23656 |
| 27 | BA101431 | Bone | 6 | 968 | 18,140 ± 80 | 22,275-21761 |
| 28 | BA101433 | Charcoal | 6 | 964 | 20,410 ± 80 | 24,910-24255 |
| 29 | BA101434 | Bone | 6 | 964 | 19,850 ± 80 | 24,139-23629 |
| 30 | BA121954 | Tooth | 6 | 961.5 | 20,155 ± 45 | 24,420-24016 |
| 31 | BA10487 | Charcoal | 6 | 1004 | 20,500 ± 100 | 25,066-24337 |
| 32 | BA10488 | Bone | 6 | 1004 | 18,090 ± 70 | 22,191-21682 |
| 33 | BA121951 | Bone | 6 | 1004 | 18,280 ± 45 | 22,342-21927 |
| 34[^d^](#bookmark4) | BA101436 | Bone | 6 | 1025 | 13,250 ± 60 | 16,137-15723 |
| 35 | BA101438 | Charcoal | 6 | 1026 | 20,350 ± 90 | 24,835-24150 |
| 36 | BA121960 | Bone | 7 | 1160 | 21,690 ± 80 | 26,100-25789 |
| 37 | BA101439 | Bone | 7 | 1160 | 19,650 ± 80 | 23,945-23414 |
| 38 | BA101442 | Charcoal | 7 | 1160 | 20,010 ± 70 | 24,302-23850 |
| 39[^d^](#bookmark4) | BA101441 | Charcoal | 7 | 1250 | 12,540 ± 70 | 15,136-14353 |
| 40 | BA101445 | Bone | 7-8 boundary | 1355 | 20,510 ± 90 | 25,056-24363 |
| 41 | BA101444 | Charcoal | 8 | 1425 | 24,185 ± 90 | 28,524-27925 |

BA = Radiocarbon Laboratory, Peking University.

^a^ Note 1: Depth is centimeters below the datum of the excavations.

^b^ Note 2: Half-life of the ^14^C is 5568 years; BP means date before 1950.

^c^ Note 3: Calibration done using OxCal 4.2 (<https://c14.arch.ox.ac.uk/oxcal.html>) and IntCal13 (Reimer et al., 2013).

^d^ Dates considered to be outliers
