# Supplementary material for: Unveiling bast fiber production in Upper Paleolithic North China: Microfibers and usewear traces on stone tools from Shizitan
Source: PLoS One. 2026 Apr 13;21(4):e0346767. doi: 10.1371/journal.pone.0346767 (PMC13075717; doi:10.1371/journal.pone.0346767)
Supplement: S2 Table — (DOCX) [file pone.0346767.s008.docx]

**S2 Table. Radiocarbon dates by layer from Shizitan 14 [5].**

|  | **Lab No.** | **Material** | **Layer** | **Depth (cm)**[**^a^**](#bookmark1) | **^14^C age (yr BP)**[**^b^**](#bookmark2) | **Calibrated age**[**^c^**](#bookmark3) **(yr BP, 95.4%)** |
| --- | --- | --- | --- | --- | --- | --- |
|  | BA101589 | Burned bone | 2 | 71 cm | 15030±150 | 18611-17901 |
|  | BA101158 | Burned bone | 3 | 75 cm | 17210±290 | 21150-19550 |
|  | BA101591 | Burned bone | 4 | 85 cm | 19050±80 | 23021-22353 |
