# Supplementary material for: Unveiling bast fiber production in Upper Paleolithic North China: Microfibers and usewear traces on stone tools from Shizitan
Source: PLoS One. 2026 Apr 13;21(4):e0346767. doi: 10.1371/journal.pone.0346767 (PMC13075717; doi:10.1371/journal.pone.0346767)
Supplement: S3 Table — (DOCX) [file pone.0346767.s009.docx]

**S3 Table. SZT 14 residue and usewear sampling record in the Shanxi Museum, Taiyuan, 2009-2010 (recorded by XC Chen and L Liu).**

| **Artifact** | **Description** | **Lithic** | **Residue sampling** | **Notes** |
| --- | --- | --- | --- | --- |
| S14 GS1  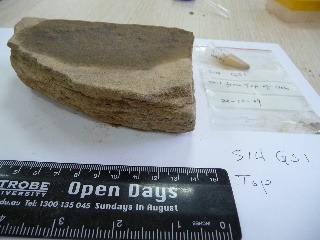 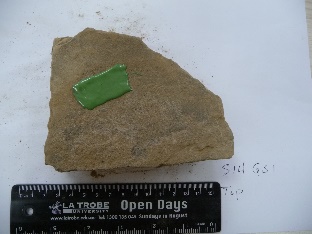 | Broken slab, ¼ remaining. Used surface slightly concave, showing pecking under magnification. The bottom side is flat with white residue sediments. Layered sandstone, fine grained. Manufacturing method: unifacially flaked; bottom side is smaller than the top side. Remaining length: 13 cm; width: 9.2 cm; thickness: 3 cm; weight: 741 g. | fine-grain purple sandstone | 2 water samples, from top and bottom; 1 soil sediment sample from top; Wet area is where residue sample was taken; 2 usewear PVS from top and bottom (green peel) | Unearthed from a location with height of 73.5 cm, on 14 May 2005; washed before sampling; Recorded in the Shanxi Museum, Taiyuan, 20 Oct. 2009 |
| S14 GS2  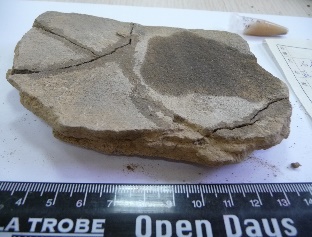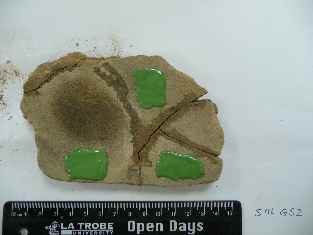 | Broken slab, remaining shape near rectangular. Used surface is concave, rough; bottom side is weathered severely. One side is straight, while other three sides are irregular. It is broken into three pieces, but glued together. Under magnification can see rough surface after use. Remaining length: 13.3 cm; width: 8.4 cm; thickness: 1.5 cm; weight: 250 g. | fine-grain gray sandstone | 1 sediment sample from top; 2 water (A, B) from top; 1 water from bottom (C); Wet area is where residue sample was taken; 3 usewear PVS from top (green peels) | Unearthed on 14 May 2005; washed and restored with glue before sampling;  Recorded in the Shanxi Museum, Taiyuan, 20 Oct. 2009 |
| S14GS3  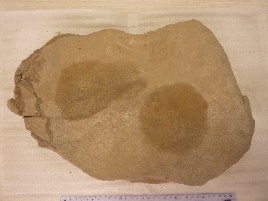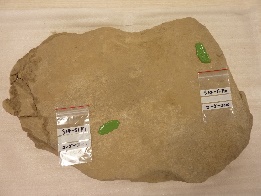 | Slab with a heavily weathered surface; an irregular shape, measuring 40 cm in length, 28 cm in maximum width, 3.4-4 cm in thickness; the top surface is concave, suggesting long time use; the bottom is uneven and heavily weathered. | Gray sandstone | 2 water samples from top; 1 water sample from bottom; 2 usewear PVS from top (green peels); 1 usewear PVS from bottom | Unearthed on 14 May, 2005;  Unwashed before sampling  Recorded in the Shanxi Museum, Taiyuan, Feb. 7, 2010 |
