# Supplementary material for: Unveiling bast fiber production in Upper Paleolithic North China: Microfibers and usewear traces on stone tools from Shizitan
Source: PLoS One. 2026 Apr 13;21(4):e0346767. doi: 10.1371/journal.pone.0346767 (PMC13075717; doi:10.1371/journal.pone.0346767)
Supplement: S4 Table — (DOCX) [file pone.0346767.s010.docx]

**S4 Table. Usewear record of SZT29 tools.**

| **Sample no**  **Artifact no** | **Strata** | **Polish** | **Striation** | **Edge rounding** | **Pitting** | **Possible function** |
| --- | --- | --- | --- | --- | --- | --- |
| **GS1, slab**  **(13699)** | 8 | low-level polish, very small and isolated polish spots | no | some | unclear | processing soft materials, used infrequently |
| **GS2, slab**  **(13805)** | 8 | very low-level polish, but slightly reticulated, flat surface | no |  | unclear | processing soft materials, used infrequently |
| **GS3, slab**  **(13110)** | 7 | P1 (top): low level, isolated;  P2 (bottom): mostly raw crystals, some isolated grains show small polished spots | P1: no; P2: parallel, short on some small polished areas | rounded or angular | no | processing mostly soft materials, but also hard minerals, both sides used; hematite on P2 |
| **GS4, slab**  **(13108)** | 7 | Low to medium polish, some reticulate, | mostly no, but some short and wide parallel striations on two spots | some rounded | present | processing soft and relatively hard materials; probably pounding |
| **GS5, slab**  **(collected)** | 7 | P1 (top): low level, isolated; P2 (top): medium level, mostly isolated, but some reticulate; P3 (bottom): raw crystals | P1: mostly no, but deep striations and fractures on some crystals; P2: some short and long; | rounded or angular | no | processing soft materials; hematite on top side; unused on bottom side |
| **GS6, slab**  **(66-109)** | 7 | P1: medium level, relatively reticulated;  P2: medium to high level, reticulated | no | some | unclear | processing very soft materials |
| **GS7, Handstone**  **(11959)** | 7 | P1: from the flat end; high-level polish, reticulated;  P2: a lateral side; high-level polish; very reticulated;  P3: an area with a part smooth and a part rough on a lateral side; high-level polish, very reticulated on the flat area; but only raw crystals exposed on the rough area, likely removed by pounding hard material; | no | common | present | tool end as polisher or grinder; lateral side P2 and P3 as grinder and hammer stone; hematite on P3 |
| **GS8.1-4, slab**  **(8396, 8397, 8398, 8399)** | 4 | all show medium level polish on isolated spots | 8397: parallel fine striations; others: no | common | present | processing mostly soft materials |
| **GS9, slab**  **(5744)** | 4 | P1: low level, mostly isolated; P2: medium level, more reticulate than P1 | P1: no; P2: occasionally fine and short striations | unclear | unclear | processing mostly soft materials |
| **GS10, slab**  **(6082)** | 4 | low-level very isolated polish spots | no | some | unclear | processing soft materials, used infrequently |
| **GS11, elongate slab**  **(3992)** | 2 | P1: from one lateral side, low to medium level, isolated;  P2: from another lateral side, no clear used traces | no | some | unclear | processing soft materials, used infrequently |
| **GS12, slab**  **(3091)** | 2 | some very small polished spots, many raw crystals | no |  | no | processing soft materials, used infrequently |
| **GS13, slab**  **(91-101-2)** | 2 | medium level isolated polish areas | no | common | unclear | processing soft materials |
| **GS14, slab**  **(2H42_81-103-2)** | 2 | very few small polished spots, mostly fresh crystals | no | some | unclear | used infrequently |
| **MB1 Chert**  **(62-105)** | 7 | high polish, reticulate on edge, flat, more extensive on P1 than P2 | unclear | unclear |  | used |
| **MB2 chert**  **(12455)** | 7 | high polish, reticulate, flat, on both sides | unclear | unclear |  | used |
| **MB3 chert**  **(13119)** | 7 | high polish, large areas, on both sides | no | unclear |  | used |
| **MB4 chert**  **(13259)** | 7 | high polish, reticulate, flat on P1 | long, parallel, and fine striations nearly horizontal to the edge on several areas on P1 | slightly |  | comparable to bone-working traces |
| **MB5 chert**  **(11491)** | 7 | high polish; less extensive on P2 than P1 | fine striations, horizontally with some diagonal on P1 | present |  | cutting plants |
| **MB6 chert**  **(60-100-1)** | 7 | P1: high polish | P1: long and wide striations, diagonal | present |  | used |
| **MB7 chert**  **(11703)** | 7 | P1: very high polish, but uneven;  P2: high polish, reticulate | P1: shallow and wide parallel striations, long and diagonal; P2: long and wide parallel striations, multidirectional, | present |  | used |
| **MB8 chert (10694)** | 6 | a lot of polish, flat surface, on the ventral side | no | no |  | hard materials |
| **SF1 quartzite scraper (13678)** | 8 | no | wide striations, diagonal, on both sides |  |  | scraping hard material |
| **SF2 quartzite scraper (74-97)** | 7 | small polished area, rarely seen, on P2 | no | no |  | used |
| **SF3 quartzite scraper**  **(79-104-3)** | 6 | all raw crystals on both sides | no | no |  | used |
| **SF4 quartzite flake (8776)** | 5 | few spots of polish on distal edge | fine striations parallel to the edge | unclear |  | cutting plants |
| **SF5 quartzite scraper (8829)** | 5 | high polish, reticulate | very fine striations, both horizontal and vertical in orientation | present |  | cutting plants |
| **SF6 quartzite scraper (8367)** | 4 | some high polish | no | present |  | cutting plants |
| **SF7 quartzite scraper (91-95-3)** | 3 | smooth surface, high polish on P1 | no | some |  | soft materials incl. plants |
| **SF8 chert scraper**  **(4230)** | 2 | high polish, reticulate on both sides | fine striations, multidirectional on both sides | present |  | cutting plants |
| **SF9 (13834)** | 8 |  |  |  |  | non-tool |
| **SF10 quartzite flake (13819)** | 8 | many polished areas on both sides, Side A shows less polish than Side B | striations multi-directional to the edge | present |  | cutting and scraping plants |
| **SF11 quartzite flake (13207)** | 7 | mostly fractures, only one spot of polish on Side B | no | unclear |  | unclear |
| **SF12 chert flake (13246)** | 7 | high polish on the edge | no | present |  | soft materials incl. plants |
| **SF13 chert flake (10731)** | 6 |  |  |  |  | non-tool |
| **SF14 quartzite flake (8777)** | 5 | very little visible polish | no | no |  | soft materials incl. plants |
| **SF15 chert flake (8825)** | 5 | few medium level polished areas | no | no |  | soft materials incl. plants |
| **SF16 quartzite flake (86-109-3)** | 4 | medium level polish, more on the dorsal side than on the ventral side | no | present |  | soft materials incl. plants |
| **SF17 quartzite flake (8134)** | 4 |  |  |  |  | non-tool |
| **SF18 chert flake (4487)** | 3 | few medium level polished areas |  | unclear |  | soft materials incl. plants |
| **SF19 chert flake (92-93-4)** | 3 | high polish on both sides and the edge | horizontal striations parallel to the edge on Side A | present |  | soft and hard materials incl. plants |
| **SF20 quartzite flake (4273)** | 2 |  |  |  |  | non-tool |
| **SF21 quartzite flake (4267)** | 2 |  |  |  |  | non-tool |
| **SF22 quartzite scraper (811)** | 1 | no clear usewear found |  |  |  | unclear |
| **SF23 chert scraper (890)** | 1 | very high polish on one side | no | present |  | siliceous plants |
