# Supplementary material for: Unveiling bast fiber production in Upper Paleolithic North China: Microfibers and usewear traces on stone tools from Shizitan
Source: PLoS One. 2026 Apr 13;21(4):e0346767. doi: 10.1371/journal.pone.0346767 (PMC13075717; doi:10.1371/journal.pone.0346767)
Supplement: S5 Table — (DOCX) [file pone.0346767.s011.docx]

**S5 Table. Usewear traces on 12 SZT fiber production tools.**

| **Tool type*** | **Layer** | **Climatic Phase** | **Polish** | **Striations** | **Possible function** |
| --- | --- | --- | --- | --- | --- |
| **29-SF10**  **quartz scraper** | 8 | Phase 1:  Pre-LGM | Many medium to high polished areas | Striations multi-directional to the edge | Cutting and scraping plants |
| **29-GS1**  **sandstone slab** | 8 |  | Low polish, isolated | None | Processing soft plants |
| **29-MB5**  **chert microblade** | 7 Top | Phase 2:  Initial LGM | High polish | Fine striations, parallel and diagonal to the edge | Cutting plants |
| **29-GS3**  **sandstone slab** | 7 Top |  | Low level isolated polish | Parallel, short striations | Processing soft plants and hematite |
| **29-GS4**  **sandstone slab** | 7 Top |  | Low to medium polish, some reticulate | Few short and wide parallel striations | Processing soft plants and hard materials, pounding |
| **29-GS5**  **sandstone slab** | 7 Top |  | Low to medium isolated polish | Few deep striations and fractures, some short and long striations | Processing soft plants and hematite |
| **29-SF4 quartz flake** | 5 | Phase 3:  Late LGM | Few spots of polish | Fine striations parallel to the edge | Cutting plants |
| **29-SF5**  **quartz scraper** | 5 |  | High polish, reticulate | Very fine striations, both parallel and perpendicular to the edge | Cutting and scraping plants |
| **29-GS8 sandstone slab** | 4 |  | Medium polish, isolated | Parallel fine striations | Processing mostly soft materials |
| **14-GS3**  **sandstone slab** |  |  | Medium-level polish | Small areas of shallow striations | Processing soft plants |
| **29-GS13**  **sandstone slab** | 2-3 | Phase 4:  Post-LGM | Medium polish, isolated | None | Processing soft materials |
| **29-SF8**  **chert scraper** | 2 |  | High polish, reticulate | Fine striations multidirectional on both sides | Cutting and scraping plants |

* MB=microblade; SF=stone flake; GS=grinding stone; 29= SZT29, 14=SZT14
