# Supplementary material for: Unveiling bast fiber production in Upper Paleolithic North China: Microfibers and usewear traces on stone tools from Shizitan
Source: PLoS One. 2026 Apr 13;21(4):e0346767. doi: 10.1371/journal.pone.0346767 (PMC13075717; doi:10.1371/journal.pone.0346767)
Supplement: S6 Table — (DOCX) [file pone.0346767.s012.docx]

**S6 Table. Microfibers and other residue remains from the 12 analyzed SZT fiber production tools.**

|  | **Fiber counts** | | | | **Fiber form** | | | **Fiber coloration** | | | | | | | **Other associated elements** | | | | | |
| --- | --- | --- | --- | --- | --- | --- | --- | --- | --- | --- | --- | --- | --- | --- | --- | --- | --- | --- | --- | --- |
| **Tool (Layer of deposits)** | **bast fib-er** | **bast fiber bundle** | **fiber UNID** | **Fib-er total** | **twist-ed form** | **Fibri-llar**  **Z-twist** | **Fibri-llar**  **S-twist** | **pink** | **blue** | **black-grey** | **green** | **red** | **hema-tite pigment** | **color fiber total** | **Hema-tite powder** | **Phytolith** | **Epidermis vessel cell** | **Crystal (size in** **µm)** | **Yeast**  **(size µm)** | **Starch** |
| **Phase 1, Pre-LGM: 28,000-27,000 cal BP** | | | | | | | | | | | | | | | | | | | | |
| **29-SF10 (8)** | 5 |  | 2 | 7 | 5 | 1 |  | 1 | 1 |  |  |  |  | 2 |  |  |  |  |  | 8 |
| **29-GS1 (8)** | 31 |  | 8 | 39 | 8 | 5 |  | 1 | 2 | 2 |  | 1 |  | 6 |  | 1 reed |  |  |  | 19 |
| **Phase 2, Initial LGM: 26,000-24,000 cal BP** | | | | | | | | | | | | | | | | | | | | |
| **29-MB5 (7Top)** | 29 |  | 14 | 43 | 11 | 4 | 1 | 1 | 2 | 1 | 1 |  |  | 5 |  |  |  |  |  | 12 |
| **29-GS3 (7 Top)** | 24 |  | 2 | 26 | 2 | 4 |  | 1 |  |  |  |  | 1 | 2 | Present |  |  |  |  | 14 |
| **29-GS4 (7 Top)** | 54 | 6 | 2 | 62 | 2 | 14 |  |  | 2 |  |  |  |  | 2 |  |  |  |  |  | 4 |
| **29-GS5 (7 Top)** | 45 | 5 | 3 | 53 | 3 | 9 | 1 | 2 | 1 | 1 |  |  |  | 4 | Present |  |  |  |  | 13 |
| **Phase 3, Late LGM: 24,000-19,500 cal BP** | | | | | | | | | | | | | | | |  |  |  |  |  |
| **29-SF4 (5)** | 6 |  |  | 6 | 1 | 2 | 1 |  |  |  |  |  |  |  |  |  |  | 5 (9.51-11.57) |  | 116 |
| **29-SF5 (5)** | 34 |  | 7 | 40 | 7 | 2 | 1 | 1 | 3 |  |  |  |  | 4 |  |  |  |  |  | 115 |
| **29-GS8 (4)** | 42 | 3 | 9 | 54 | 9 | 3 | ?? |  | 4 | 2 |  |  |  | 6 |  |  |  |  |  | 107 |
| **14-GS3** | 76 | 6 | 3 | 85 | 7 | 18 | 2 | 2 | 2 | 1 |  |  |  | 5 |  | 4 curved hair cells | 23  epidermis;  6 pitted vessel cells | 10 (5.11-26.86) | 4 (6.99-10.57) | 118 |
| **Phase 4, Post-LGM: 19,000-18,000 cal BP** | | | | | | | | | | | | | | | | | | | | |
| **29-SF8 (2)** | 86 | 4 | 18 | 108 | 9 | 24 |  | 1 | 3 |  |  |  |  | 4 |  |  |  |  |  | 58 |
| **29-GS13 (2)** | 13 | 1 | 9 | 23 | 8 | 1 |  | 1 | 1 | 4 |  |  |  | 6 |  |  |  |  |  | 13 |
| **Total n.** | 445 | 25 | 77 | 546 | 72 | 87 | 5 | 11 | 21 | 11 | 1 | 1 | 1 | 46 | Present | 10 | 29 | 15 | 4 | 597 |
| **Total %** | 81.5% | 4.6% | 14.1% | 100.0% | 13.2% | 15.9% | 0.9% | 2.0% | 3.8% | 2.0% | 0.2% | 0.2% | 0.2% | 8.4% |  |  |  |  |  |  |
| **Ubiquity n.** | 12 | 6 | 11 | 12 | 12 | 12 | 4 | 9 | 10 | 6 | 1 | 1 | 1 | 11 |  |  |  |  |  |  |
| **Ubiquity %** | 100 | 50.0 | 91.7 | 100 | 100 | 100.0% | 33.3% | 75.0 | 83.3 | 50.0 | 8.3 | 8.3 | 8.3 | 91.7 |  |  |  |  |  |  |
| **Control sample** | | | | | | | | | | | | | | | | | | | | |
| **14-GS3:3 unused surface** |  |  |  | 0 |  |  |  |  |  |  |  |  |  |  |  |  |  |  |  |  |
| **29-GS8 bottom** | 19 |  | 1 | 20 |  |  |  |  |  |  |  |  |  |  |  |  |  |  |  | - |
| **29-SS1 soil** | 11 |  |  | 11 |  |  |  |  |  |  |  |  |  |  |  |  |  |  |  |  |
| **29-SS2 soil** | 14 |  |  | 15 |  |  |  |  | 1 | 2 |  |  |  |  |  |  |  |  |  |  |
| **29-SS3 soil** | 7 |  |  | 7 |  |  |  |  |  | 1 |  |  |  |  |  |  |  |  |  | - |
| **Natural sandstone** | 5 |  |  | 5 |  |  |  |  |  |  |  |  |  |  |  |  |  |  |  | - |
