# Supplementary material for: Unveiling bast fiber production in Upper Paleolithic North China: Microfibers and usewear traces on stone tools from Shizitan
Source: PLoS One. 2026 Apr 13;21(4):e0346767. doi: 10.1371/journal.pone.0346767 (PMC13075717; doi:10.1371/journal.pone.0346767)
Supplement: S7 Table — (DOCX) [file pone.0346767.s013.docx]

**S7 Table. Microfossil remains from the other 26 analyzed SZT tools.**

|  | **Fiber counts** | | | | | | **Fiber form** | | | | | **Fiber coloration** | | | | | | | | | | | | **Other associated elements** | | | | | |
| --- | --- | --- | --- | --- | --- | --- | --- | --- | --- | --- | --- | --- | --- | --- | --- | --- | --- | --- | --- | --- | --- | --- | --- | --- | --- | --- | --- | --- | --- |
| **Tool (Layer of deposits)** | **bast fiber** | **bast fiber bundle** | | **fiber UNID** | | **fiber total** | | **twisted form** | **fibrillar**  **Z-twist** | | **Fibri-llar**  **S-twist** | | **pink** | **blue** | | **black-grey** | | **green** | | **red** | **hema-tite pigment** | | **color fiber total** | **Hema-tite powder** | **Phyto-lith** | **Epid-ermis** | **Crys-tal** | **Yeast** | **Star-ch** |
| **Phase 1, Pre-LGM: 28,000-27,000 cal BP** | | | | | | | | | | | | | | | | | | | | | | | | | | | | | |
| **29-SF1 (8)** | 3 |  |  | | 3 | | 1 | | 2 | 1 | |  | | |  | |  | |  |  |  |  | |  |  |  |  |  | 5 |
| **29-GS2 (8)** | 8 |  | 12 | | 20 | | 10 | | 4 |  | |  | | |  | |  | |  |  |  |  | |  | 1 Reed |  |  |  | 12 |
| **Phase 2, Intial LGM: 26,000-24,000 cal BP** | | | | | | | | | | | | | | | | | | | | | | | | | | | | | |
| **29-MB1**  **(7 Top)** | 1 |  | 1 | | 2 | | 2 | |  | 1 | | 1 | | |  | |  | |  |  |  | 1 | |  |  |  |  |  | 4 |
| **29-MB2**  **(7 Top)** | 3 |  |  | | 3 | | 1 | | 3 |  | |  | | |  | |  | |  |  |  |  | |  |  |  |  |  | 1 |
| **29-MB3**  **(7 Top)** | 3 |  | 1 | | 4 | | 1 | | 3 |  | | 2 | | |  | |  | |  |  |  | 2 | |  |  |  |  |  | 0 |
| **29-MB4**  **(7 Top)** | 4 |  | 3 | | 7 | | 2 | | 3 |  | |  | | |  | |  | |  |  |  |  | |  |  |  |  |  | 4 |
| **29-MB6**  **(7 Top)** | 2 |  | 2 | | 4 | | 1 | | 3 | 2 | |  | | |  | |  | |  |  |  |  | |  |  |  |  |  | 0 |
| **29-SF2**  **(7 Top)** | 3 |  | 1 | | 4 | |  | | 3 |  | |  | | |  | | 1 | |  |  |  | 1 | |  |  |  |  |  | 19 |
| **29-SF11**  **(7 Top)** | 13 |  | 1 | | 14 | | 1 | | 1 |  | | 1 | | | 1 | |  | |  |  |  | 2 | |  |  |  |  |  | 14 |
| **29-SF12**  **(7 Top)** | 7 |  | 1 | | 8 | | 4 | | 1 |  | |  | | |  | | 1 | |  |  |  |  | |  |  |  |  |  | 2 |
| **29-GS6**  **(7 Top)** | 14 | 1 | 2 | | 17 | | 4 | | 4 |  | | 2 | | | 2 | |  | |  |  |  | 4 | |  |  |  |  |  | 3 |
| **29-GS7**  **(7 Top)** | 7 |  | 7 | | 14 | | 3 | | 3 |  | |  | | |  | | 1 | |  |  |  | 1 | | present |  |  |  |  | 46 |
| **Phase 3, Late LGM: 24,000-19,500 cal BP** | | | | | | | | | | | | | | | | | | | | | | | | | | | | | |
| **29-MB8 (6)** | 8 |  | 3 | | 11 | | 4 | | 6 | 1 | | 1 | | |  | |  | |  |  |  | 1 | |  |  |  |  |  | 4 |
| **29-SF13 (6)** | 19 |  | 3 | | 22 | | 2 | | 1 | 2 | | 2 | | | 1 | | 2 | |  |  |  | 5 | |  |  |  |  |  | 0 |
| **29-SF14 (5)** | 23 | 2 | 2 | | 27 | | 3 | | 7 |  | | 1 | | | 2 | | 4 | |  | 1 |  | 7 | |  |  |  |  |  | 11 |
| **29-SF15 (5)** | 15 | 2 |  | | 17 | | 3 | | 4 | 2 | |  | | | 2 | |  | |  |  |  | 2 | |  |  |  |  |  | 10 |
| **29-SF16 (4)** | 4 |  | 5 | | 9 | |  | | 1 |  | |  | | |  | |  | |  |  |  |  | |  |  |  |  |  | 4 |
| **29-SF17 (4)** | 10 | 1 | 1 | | 12 | | 3 | |  |  | |  | | |  | |  | |  |  |  |  | |  |  |  |  |  | 0 |
| **29-SF6 (4)** | 8 |  |  | | 8 | | 1 | | 4 |  | |  | | |  | |  | |  |  |  |  | |  |  |  |  |  | 142 |
| **14-GS1** | 6 |  |  | | 6 | |  | |  |  | |  | | |  | |  | |  |  |  |  | |  |  |  |  |  | 6 |
| **14-GS2** | 6 | 1 |  | | 7 | |  | |  |  | |  | | |  | |  | |  |  |  |  | |  |  |  |  |  | 4 |
| **Phase 4, Post-LGM: 19,000-18,000 cal BP** | | | | | | | | | | | | | | | | | | | | | | | | | | | | | |
| **29-SF18 (3)** | 2 |  |  | | 2 | |  | | 3 |  | |  | | |  | |  | |  |  |  |  | |  |  |  |  |  | 6 |
| **29-SF19 (3)** | 7 |  |  | | 7 | |  | | 6 |  | |  | | |  | |  | |  |  |  |  | |  |  |  |  |  | 4 |
| **29-SF7 (3)** | 5 |  |  | | 5 | | 1 | | 1 |  | |  | | |  | | 1 | |  |  |  | 1 | |  |  |  |  |  | 120 |
| **29-SF20 (2)** | 6 |  |  | | 6 | | 1 | |  |  | |  | | |  | |  | |  |  |  |  | |  |  |  |  |  | 0 |
| **29-SF21 (2)** | 10 |  | 5 | | 15 | | 1 | | 1 |  | | 4 | | |  | | 2 | |  |  |  | 6 | |  |  |  |  |  | 0 |
| **Total n.** | 197 | 7 | 49 | | 254 | | 49 | | 64 | 9 | | 14 | | | 8 | | 12 | |  | 1 |  | 32 | |  |  |  |  |  | 421 |
| **Total %** | 77.6% | 2.8% | 19.3% | | 100.0% | | 19.3% | | 25.2% | 3.5% | | 5.5% | | | 3.1% | | 4.7% | | 0.0% | 0.4% | 0.0% | 12.6% | |  |  |  |  |  |  |
| **Ubiquity n.** | 26 | 5 | 15 | | 26 | | 20 | | 21 | 6 | | 8 | | | 5 | | 7 | | 0 | 1 | 0 | 11 | | 1 | 1 | 0 | 0 | 0 | 26 |
| **Ubiquity %** | 100.0% | 19.2% | 57.7% | | 100.0% | | 76.9% | | 80.8% | 23.1% | | 30.8% | | | 19.2% | | 26.9% | | 0.0% | 3.8% | 0.0% | 42.3% | | 3.8 | 3.8 | 0.0 | 0.0 | 0.0 | 100 |
