# Supplementary material for: Unveiling bast fiber production in Upper Paleolithic North China: Microfibers and usewear traces on stone tools from Shizitan
Source: PLoS One. 2026 Apr 13;21(4):e0346767. doi: 10.1371/journal.pone.0346767 (PMC13075717; doi:10.1371/journal.pone.0346767)
Supplement: S8 Table — (DOCX) [file pone.0346767.s014.docx]

**S8 Table. Ethnographic studies of the hemp production process.**

| **Activity, environment, and tools** | **South Korea** | **The Hmong in Vietnam** | **Tai’an, Shandong, China** | **Zhongmou, central Henan, China** | **Yanshi, central Henan, China** | **Xichuan, south Henan, China** | **Yichuan, North Shaanxi, China** | **Tongde, Qinghai, China** |
| --- | --- | --- | --- | --- | --- | --- | --- | --- |
| **Fibrous plants** | Hemp | Hemp | Hemp | Hemp, velvetleaf | Velvetleaf | Velvetleaf, ramie | Hemp | Hemp |
| **Products** | Textiles and other fiber products | Textiles and other fiber products | Crude ropes and other fiber products, ribbons sold to textile mill | String and rope | Rope | String and rope | Strings and rope | String |
| **Harvesting tools** | Sickle and knife | Sickle | Sickle and knife | Sickle | Uproot plant with pick and chop off roots with axe | Sickle | Sickle | Sickle |
| **Retting** | Water retting | Dew retting | Water retting | Water retting | Water retting | Water retting | Water retting | Water retting |
| **Decortication** | By hand | By hand | By hand | By hand | By hand | By hand | By hand | By hand |
| **Pounding with**  **stone or wooden tools** | In Gokseong, pounded with rocks or treaded under foot to soften the fiber | Fiber bunches pounded in a mortar with a wooden pestle | No | No | No | No | No | No |
| **Scraping with**  **scraper** | In Andong, the wetted bark strips are scraped with a blunt knife against a wooden anvil | Not mentioned | No | No | No | No | No | No |
| **Splitting and splicing** | Both, manually | Both, manually | No | Both, manually | Both, manually | Both, manually | Both, manually | Both, manually |
| **Making yarn** | Additional twist, using a wooden, hand-operated, single-spindle spinning wheel | Additional twist, using a large wheel turned by foot | Small stalks used directly for binding without any further processing | Twisting manually for making strings, using simple equipment for making ropes | Using simple equipment for making ropes | Twisting manually for making strings, using simple equipment for making ropes | Twisting manually for making strings, using simple equipment for making ropes | Twisting manually for making strings |
| **Weaving** | Using loom | Using loom | N/A | N/A | N/A | N/A | N/A | N/A |
| **References** | [29] | [30] | [31] | Personal comm.  WU Haoye, GUO Fengrong, and ZHANG Xiurong, 2024 | Personal comm.  WANG Facheng, 2024 | Personal comm.  LIU Zhongwei, 2024 | Personal comm. YE Wa and WEI Jinchuan, 2024 | Personal comm. SHI Lanying and Geriduojie, 2024 |
