# Supplementary material for: Unveiling bast fiber production in Upper Paleolithic North China: Microfibers and usewear traces on stone tools from Shizitan
Source: PLoS One. 2026 Apr 13;21(4):e0346767. doi: 10.1371/journal.pone.0346767 (PMC13075717; doi:10.1371/journal.pone.0346767)
Supplement: S9 Table — (DOCX) [file pone.0346767.s015.docx]

**S9 Table. Colorations of dyed fibers and possibly related dyeing plants in the Shizitan area.**

| **Dates** | **SZT29 Layer** | **Fiber colors** | **Possible dyeing plants** | **Corresponding pollen in SZT area** |
| --- | --- | --- | --- | --- |
| **Pre-LGM** | 8 | Pink | Madder (*Rubia cordifolia*) |  |
|  |  | Blue | Chinese woad (*Isatis indigotica*) |  |
|  |  | Black-grey | *Quercus* acorn cap  Goosefoot (*Chenopodium album*) | *Quercus* Chenopodiaceae |
| **Initial LGM** | 7 Top | Pink | Madder |  |
|  |  | Blue | Chinese woad |  |
|  |  | Black-grey | *Quercus* acorn cap  Goosefoot | Chenopodiaceae |
|  |  | Green | Dahurian buckthorn (*Rhamnus davurica*) |  |
| **Late LGM-Post LGM** | 2-6 | Pink | Madder |  |
|  |  | Blue | Chinese woad |  |
|  |  | Black-grey | *Quercus* acorn cap  Goosefoot | Chenopodiaceae |
|  |  | Red | Madder |  |
| **Corresponding plants in Shanxi today** | Early Holocene, Modern | Pink | Madder |  |
|  |  | Blue | Chinese woad | Cruciferae |
|  |  | Black-grey | *Quercus* acorn cap, Goosefoot |  |
|  |  | Green | Walnut, Dahurian buckthorn |  |
|  |  | Red | Madder |  |
