# Supplementary material for: Unveiling bast fiber production in Upper Paleolithic North China: Microfibers and usewear traces on stone tools from Shizitan
Source: PLoS One. 2026 Apr 13;21(4):e0346767. doi: 10.1371/journal.pone.0346767 (PMC13075717; doi:10.1371/journal.pone.0346767)
Supplement: S10 Table — (DOCX) [file pone.0346767.s016.docx]

**S10 Table. Seasonality of plants related to fiber and food production available in Shanxi.**

| **Common name** | **Chinese term** | **Scientific name** | **Harvesting time** | **References** |
| --- | --- | --- | --- | --- |
| ***Fibrous plants*** | | | | |
| **hemp** | *dama* 大麻 | *Cannabis sativa* | Mid-June | [32, 33] |
| **wild flax** | *yama* 亚麻 | *Linum stelleroides* | June | [34] |
| **velvetleaf** | *qingma* 苘麻 | *Abutilon theophrastii* | July-Aug. | [33, 34] |
| ***Dyeing plants for colors*** | | | | |
| **Madder for different shades of red** | *qiancao* 茜草 | *Rubia cordifolia* | May-Sept., better quality in Sept. | [33, 35] |
| **Chinese woad for blue** | *songlan* 菘蓝 | *Isatis indigotica* | June-Oct | [33, 36] |
| **acorn caps for black** | *zaodou* 皂斗 | *Quercus* spp. | Sept.-Oct. | [33, 37] |
| **Goosefoot for grey** | *li* 藜*, huihuicai* 灰灰菜 | *Chenopodium album* | May-Oct. | [37] |
| **green husk of walnut for green** | *hetao qingpi* 核桃青皮 | *Juglans* spp. | Sept. | [37] |
| **dahurian buckthorn for green** | *shuli* 鼠李 | *Rhamnus davurica* | Aug.-Sept. | [33, 34] |
| ***Food plants*** | | | | |
| **green foxtail** | *gouweicao* 狗尾草 | *Setaria viridis* | May-Oct. | [38] |
| **Triticeae** | *xiaomaizu* 小麦族 | Triticeae | June-Sept. mostly July-Sept. | [38] |
| **Job’s tears** | *yiyi* 薏苡 | *Coix lacryma-jobi* L. | Sept.-Oct. | [38] |
| **snake gourd root** | *gualougen* 栝楼根 | *Trichosanthes kirilowii* | Nov. fully mature | [35] |
| **lily** | *baihe* 百合 | *Lilium* spp. | Aug.-Oct. | [38] |
| **yam** | *shanyao* 山药 | *Dioscorea* spp. | Oct.-Nov. | [38] |
